# Biochemical and Mechanistic Insights into O_2_ Scavenging and Stability of the Soluble Hydrogenase from *Hydrogenophaga pseudoflava*

**DOI:** 10.1021/acsomega.5c09877

**Published:** 2025-12-30

**Authors:** Dominik L. Siebert, Simon Grieshaber, Bastian Blombach, Volker Sieber, Ammar Al-Shameri

**Affiliations:** a Chair of Chemistry of Biogenic Resources, TUM Campus Straubing for Biotechnology and Sustainability, Technical University of Munich, Schulgasse 16, Straubing 94315, Germany; b Microbial Biotechnology, TUM Campus Straubing for Biotechnology and Sustainability, 198054 Technical University of Munich, Uferstraße 53, Straubing 94315, Germany; c Catalytic Research Center, 9184 Technical University of Munich, Garching 85748, Germany

## Abstract

Bidirectional hydrogenases are unique enzymes capable of regenerating costly cofactors such as NAD­(P)H using H_2_, making them highly attractive for biotechnological applications. The most studied example, the soluble hydrogenase from*Cupriavidus necator*­(*Cn*SH), is limited by low stability, and poor activity for cofactors beyond NAD^+^. In this study, we produced, purified, and characterized the soluble hydrogenase from the aerobic carboxydotrophic Knallgas bacterium*Hydrogenophaga pseudoflava*­(*Hp*SH) and benchmarked it against *Cn*SH. *Hp*SH exhibits a broader cofactor spectrum than *Cn*SH, including H_2_-driven reduction of NADP^+^, FMN, and FAD. It also demonstrates superior stability, retaining its activity for over 72 h at 30 °C. We investigated the effect of O_2_ on *Hp*SH activity and found that it is an O_2_-tolerant enzyme, as it catalytically scavenges O_2_ before initiating the reduction of the cofactor. This reactivation mechanism is distinct, requiring both H_2_ and NADH for effective O_2_ detoxification. We validated the utility of *Hp*SH in cofactor regeneration, achieving a total turnover number (TTN) of 2.23 × 10^5^, underscoring its promise for biotechnological applications, particularly in microaerobic and anaerobic settings.

## Graphical abstract

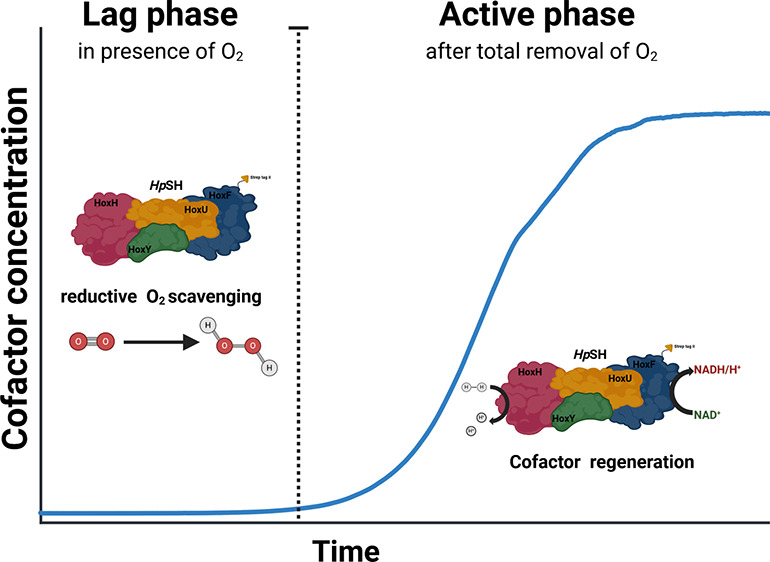

## Introduction

Molecular hydrogen (H_2_) is a clean, high-energy reductant that, when harnessed by hydrogenases, can drive a wide range of biochemical reactions. Hydrogenases catalyze the reversible oxidation of H_2_ to protons and electrons (H_2_ ⇌ 2H^+^ + 2e^–^) using a metallocofactor.^1,2^ They are classified by the type of metallic cofactor bound to their active site into three unrelated groups of [Fe],^3^ [FeFe],^4^ and [NiFe]-hydrogenases.^5^ Unlike [FeFe] hydrogenases, which are oxygen sensitive, some [NiFe]-hydrogenases show a tolerance toward oxygen, enabling them to remain catalytically active in the presence of O_2_.^6–9^ Among the [NiFe]-hydrogenases, the bidirectional soluble hydrogenase from *Cupriavidus necator (CnSH*) has become a pillar for H_2_-driven NAD­(P)^+^/NAD­(P)H and flavin cofactor recycling.^10–12^ *CnSH* has been extensively studied and thoroughly characterized both biochemically and spectroscopically.^13^ *Cn*SH is a hexameric complex, consisting of the subunits HoxFUYHI_2_, which comprises a hydrogenase module (HoxYH), a diaphorase module (HoxFU), and a regulatory HoxI dimer.^14^ Electrons from H_2_ oxidation at the Ni–Fe active site travel through iron–sulfur clusters and FMN to reduce NAD^+^ in the diaphorase module.^15^ *Cn*SH and its close relatives are of high interest for biotechnological applications, not only for hydrogen cofactor recycling, but also for NADH oxidation and hydrogen production.^16^ Recently, a set of annotated genes from the betaproteobacteria *Hydrogenophaga pseudoflava* DSM 1084 exhibited conserved binding regions for Ni–Fe, FMN, and NADP^+^ cofactors as well as iron–sulfur clusters (Table S2, Supporting Information Appendix). This suggests that this enzyme belongs to the [NiFe] Group 3 hydrogenases, also known as the cofactor-coupled bidirectional [NiFe]-hydrogenases.^17^ The annotated genes revealed that four genes (HoxHYFU) of *H. pseudoflava* show homology to the structural subunits of *Cn*SH (Figure 1).^18^ Similarly, *hoxW*, which encodes the endopeptidase HoxW required for HoxH maturation. HoxW cleaves HoxH at a specific site located at its C-terminus. This also exhibits homology to *Cn*SH, in particular, a conserved region around the cutting site. Interestingly, there are multiple studies on the membrane-bound [NiFe]-hydrogenase extracted from different strains of *H. pseudoflava*.^19,20^ However, there is still no detailed information available about its soluble hydrogenase. *H. pseudoflava* is an aerobic carboxydothrophic Knallgasbacterium.^21^ Thus, its soluble hydrogenase (*Hp*SH) may also be oxygen-tolerant. In this work, we first establish a homologous overexpression and purification system for *Hp*SH, then perform comprehensive biochemical profiling while comparing it to available data for *Cn*SH. Finally, we elucidate the origin and mechanism of its characteristic lag phase and demonstrate the efficacy of NADH regeneration.

**Figure 1:**
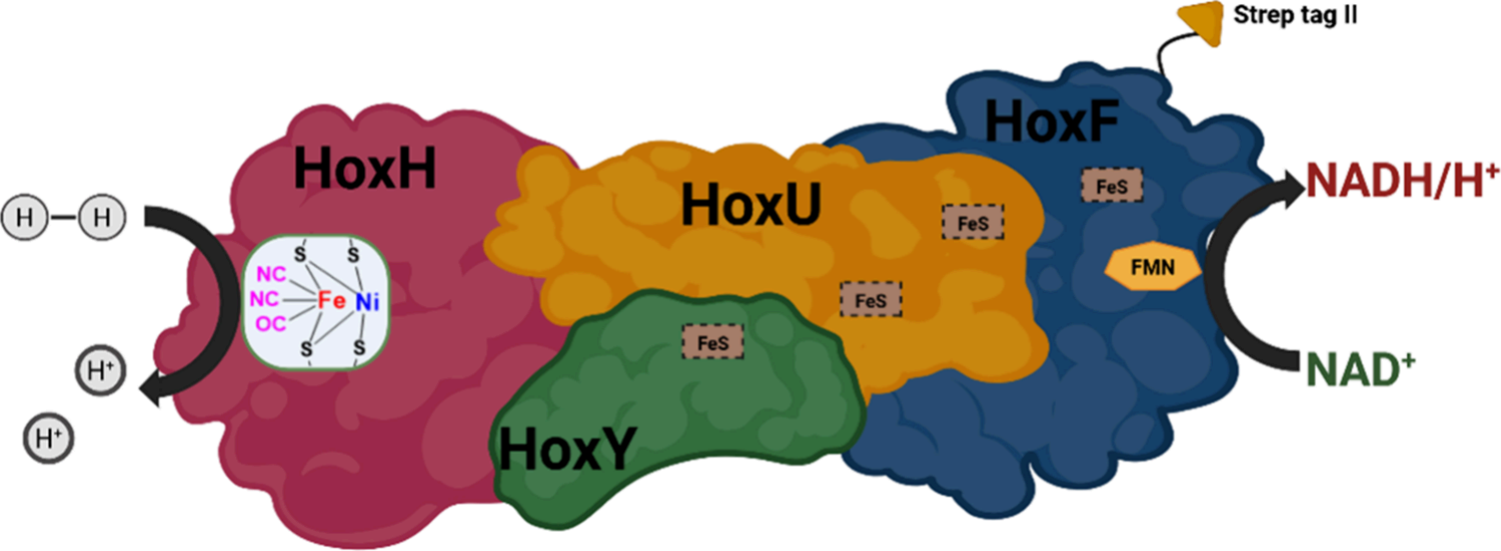
Simplified depiction of soluble [Ni–Fe] hydrogenases, also known as bidirectional hydrogenases, without HoxI subunits.

## Results

### Expression Yield and Purity

We developed an overexpression system for producing *Hp*SH as described in the method section. An operon carrying the genes encoding the structural subunits (HoxHYFU) of *Hp*SH and an endopeptidase required for HoxH maturation (HoxW*)* was introduced into its native host *via* a plasmid-based approach, as explained in detail below. Unlike *C. necator*, no HoxI gene was identified. A Strep-Tag was cloned on the N-terminus of the HoxF subunit to facilitate purification *via* affinity chromatography using Strep-Tactin matrix. The production and purification of *Hp*SH are explained in the methods section. In short, single colonies were picked and used for two stages of precultures. Main cultures were grown aerobically at 30 °C in CO-Ox medium containing 1% fructose in either 100- or 200 mL cultures with 90% headspace. Cells were harvested after reaching an OD_600_ of 4 in the late exponential phase *via* centrifugation. The enzyme was purified using the same protocol as for *Cn*SH. The purification yielded 0.67 mg L^–1^ of purified *Hp*SH containing the four expressed subunits. The SDS-PAGE of the purified *Hp*SH and its UV–vis spectrum resembled that of *Cn*SH,^22^ As shown in (Figure S1). The size of HoxH and HoxU did not exactly match the expected sizes, even considering the predicted size of the cleaved HoxH after maturation. The slightly altered flow conditions might be due to the individual amino acid composition of the subunits affecting their migration behavior within the gel. In addition, the observed bands revealed a predominant presence of HoxF compared to the other subunits. This finding aligns with our previous results on the purification of heterologously produced hydrogenases in *E. coli*. Since Strep-tag is only fused to HoxF, a part of the tetrameric enzyme might have lost part of its integrity during purification. Another possibility is that the aerobic conditions during cultivation and purification may compromise the enzyme’s quaternary structure.

### Activity of *Hp*SH and Lag Phase

Initially, the purified enzyme showed no H_2_-driven activity for NAD^+^ reduction. One plausible explanation for this might be an inactive diaphorase module. Therefore, the enzyme was incubated with methylene blue to test whether the hydrogenase module was capable of H_2_ oxidation, as methylene can accept electrons independently from the diaphorase. Initially, it was not possible to detect any reduction of methylene blue; however, when the enzyme was left with H_2_ and methylene blue overnight, the redox dye got reduced (Figure S2). The specific activity toward methylene blue was relatively low, approximately 30 mU mg^–1^. This was a strong indicator of either a low catalytic rate or a lag phase. To test whether these findings could also be applied toward the H_2_-driven NAD^+^ reduction, we elongated the activity measurement incubation. We observed a steep rise in activity after approximately 30 min (Figure 2), indicating that *Hp*SH was able to reduce NAD^+^ after a prolonged lag phase. Following this discovery, we elongated the assay time to 90 min to determine the optimal performance parameters for *Hp*SH.

**Figure 2:**
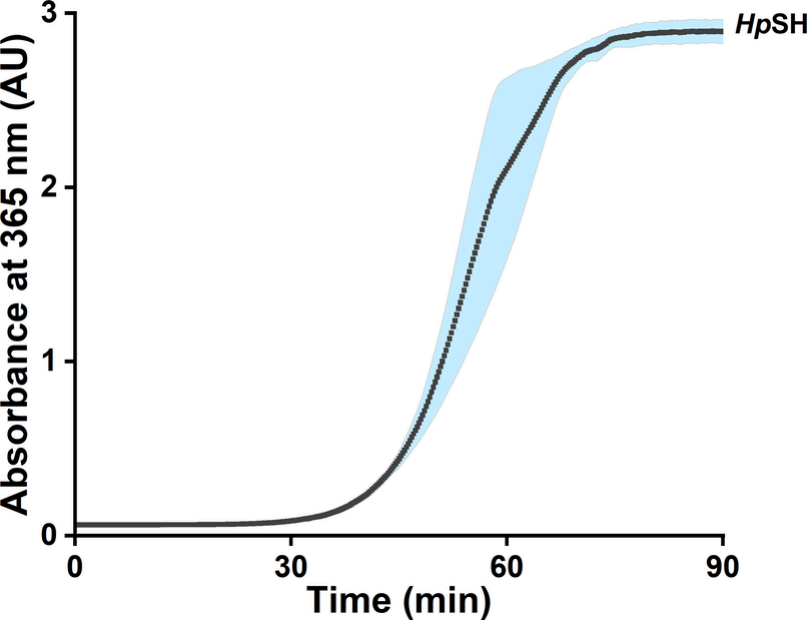
Lag phase of the purified *Hp*SH during the H_2_-driven reduction of NAD^+^. The measurement was performed at 30 °C using a H_2_-saturated reaction buffer containing (2 μg mL^–1^) of *Hp*SH, 1 mM NAD^+^, 50 mM Tris-HCl, 1 mM TCEP, and 1 μM FMN. The reaction conditions used are further defined in the methods section.

### Biochemical Profiling

Starting with the temperature, we tested the activity of *Hp*SH at temperatures from 20 to 50 °C (Figure 3A). We observed a temperature optimum at 30 °C, which is close to the optimum of *Cn*SH, with activity dropping significantly at 50 °C.

**Figure 3:**
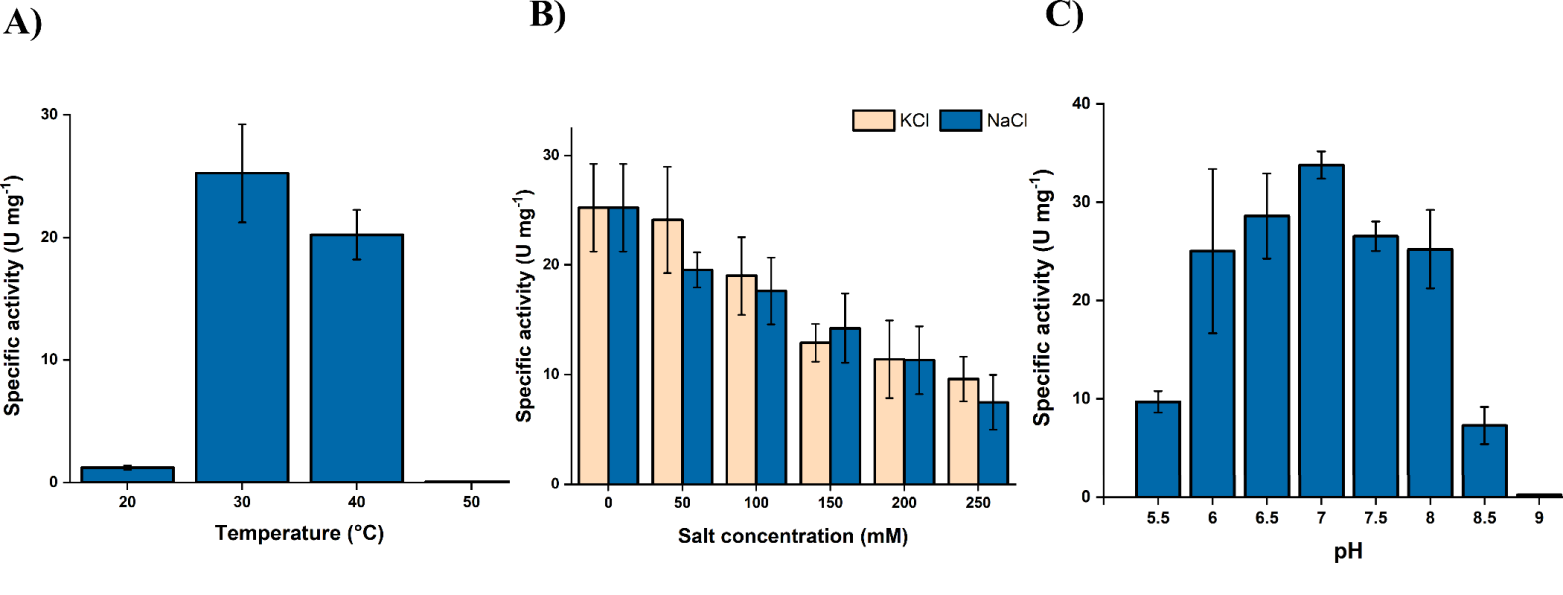
Biochemical characterization of the purified *Hp*SH (2 μg mL^–1^). The specific activity was measured as the H_2_-driven reduction of 1 mM NAD^+^, with activity profiles at different temperatures (A), KCl and NaCl concentrations (B), and pH values (C). The measurements were performed at 30 °C using a H_2_-saturated reaction buffer containing 1 mM NAD^+^, 50 mM Tris-HCl, 1 mM TCEP, and 1 μM FMN. The reaction conditions used are further defined in the methods section. For pH values below 7, the system was buffered with 50 mM MES instead of Tris-HCl.

Following this, we investigated the ionic strength stability by testing the effect of KCl and NaCl on the enzyme activity (Figure 3B). An increase in salt concentration led to a reduction in the specific activity of the enzyme, although the enzyme appears to tolerate both KCl and NaCl equally. *Hp*SH shows a higher tolerance to NaCl compared to *Cn*SH. *Hp*SH still showed 75% residual activity at 100 mM NaCl, whereas *Cn*SH only retained 30%.^23^ Regarding the pH optimum, pH values ranging from 5.5 to 9 were tested (Figure 3C). The maximum activity of 33.8 ± 1.3 U mg^–1^ was observed at neutral pH 7.0, unlike *Cn*SH, which favors a more basic pH 8.0–8.5, and loses 33% of its activity at pH 7.^24^

To evaluate the potential of *Hp*SH for cofactor recycling at neutral pH, we explored the cofactor spectrum of *Hp*SH. We tested the activity of *Hp*SH on flavin cofactors and on NADP^+^. *Hp*SH showed activities for each cofactor (Figure 4A and Figures S4 and S5). Remarkably, *Hp*SH reduced NADP^+^, with a specific activity of 0.8 U mg^–1^, surpassing native *Cn*SH and reaching up to 80% of the activity of the best NADP^+^ engineered variant of CnSH^E341A/S342R^ so far.^25^ *Hp*SH was also capable of converting both FAD and FMN with significantly higher activities than *Cn*SH, reaching up to 1.9 U mg^–1^ for FAD and 8.5 U mg^–1^ for FMN. This corresponds to a 10-fold and 1.5-fold higher activity for FAD and FMN, respectively, compared to *Cn*SH.^26^ It would also be interesting to investigate whether *Hp*SH can reduce synthetic cofactors similar to *Cn*SH, although this remains a question for future studies.^27^

**Figure 4:**
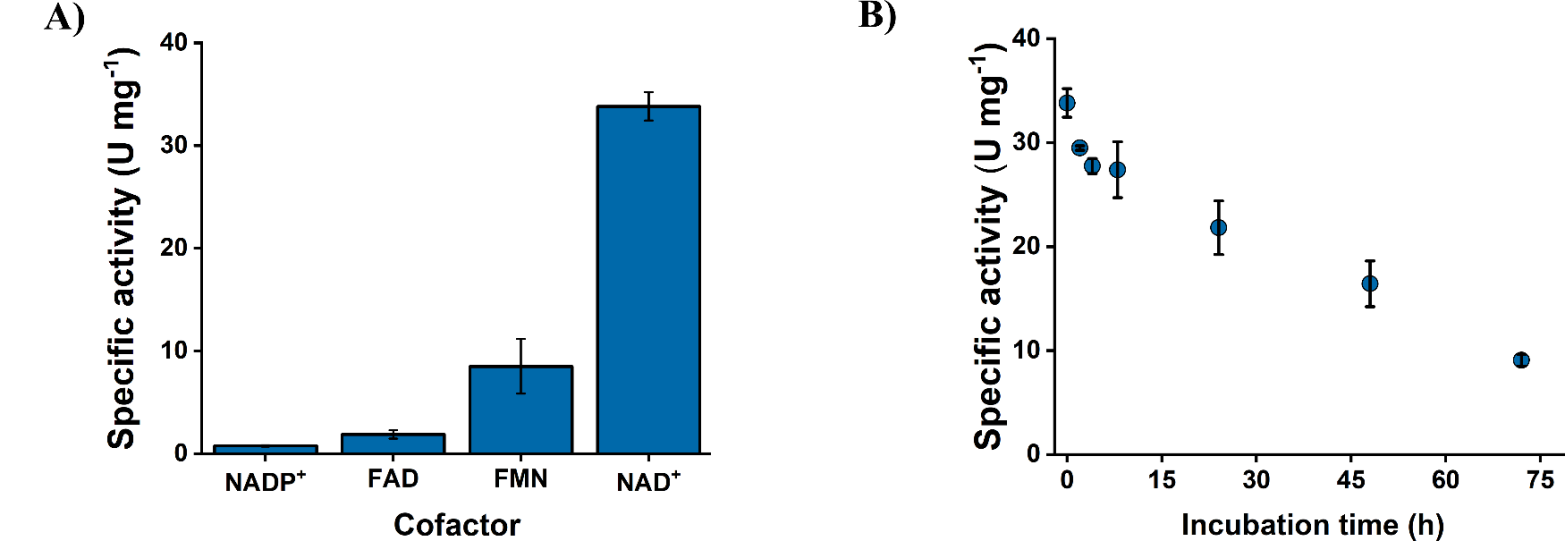
Analysis of the substrate spectrum of *Hp*SH (2 μg mL^–1^) by measuring the H_2_-driven reduction of cofactors NAD^+^, NADP^+^, FMN, and FAD at 30 °C, pH 7.0 (A). Determination of the temperature stability of *Hp*SH at 30 °C, pH 7.0, measured by H_2_- driven reduction of 1 mM NAD^+^ (B). The measurements were performed at 30 °C using a H_2_-saturated reaction buffer containing 1 mM of cofactor, 50 mM Tris-HCl, 1 mM TCEP, and 1 μM FMN. For flavin cofactors, no additional FMN was added. For temperature stability measurements, the enzyme was incubated at 30 °C for different periods of time before activity measurements. The reaction conditions used are further defined in the methods section.

Subsequently, we determined the catalytic parameters using NAD^+^ under the optimized conditions of pH 7, 50 mM Tris, no additional salt, and 30 °C. We determined a K_M_ value of 0.4 ± 0.14 mM, and a *k*_cat_ of 137 ± 20 s^–1^. This resulted in a catalytic efficiency for NAD^+^ (*k*_cat_/K_M_) of 2.3 × 10^5^ M^–1^ s^–1^, which is lower but still comparable to *Cn*SH (Figure S6 and Table S11).^25,23^

It was not possible to determine the catalytic parameters for the other cofactors, as the activity on NADP^+^ was too low and the conversion of flavin cofactors, particularly FAD, exhibited a significantly long lag phase (Table S10). Finally, we tested the stability of *Hp*SH by incubating the enzyme at 30 °C and pH 7. The enzyme exhibited a residual activity of 26% even after 72 h, with a half-life time of approximately 46 h (Figure 4B). This exceeds the half-life time of *Cn*SH of 10 h at 25 °C and only 5 h at 35 °C.^23^ It is essential to note that *Hp*SH preparations likely contain a high fraction of incompletely assembled complexes, meaning the observed activities represent apparent *k*_cat_ values, and actual activity may be higher. Improving enzyme integrity will require optimization steps such as coexpression of maturation machinery and refinement of the cultivation and purification protocol.

### Lag Phase Investigation

Interestingly, the length of the observed lag phase varied at different temperatures, pHs, and salt concentrations as seen in (Tables S1–S8). Since *Hp*SH was purified aerobically, we hypothesized that residual O_2_ would be behind the prolonged lag phase, as it has been reported for other hydrogenases. To investigate this, we tested the effect of O_2_ content on the activity and lag phase of *Hp*SH. We mixed H_2_ with an O_2_-saturated buffer at given concentrations. The presence of O_2_ seemed to influence the activity of *Hp*SH. At first glance, adding 1% (v/v) of O_2_-saturated buffer showed a decrease in the activity of *Hp*SH by around 20%; however, a further increase in O_2_ didn′t seem to escalate this effect (Figure 5A). An ANOVA test comparing the significance of the activity decrease over all measured data points, including the sample without O_2_, yielded a p-value of 0.27, indicating that the observed change is not statistically significant. Therefore, the amount of O_2_ has no apparent effect on the activity of *Hp*SH.

**Figure 5:**
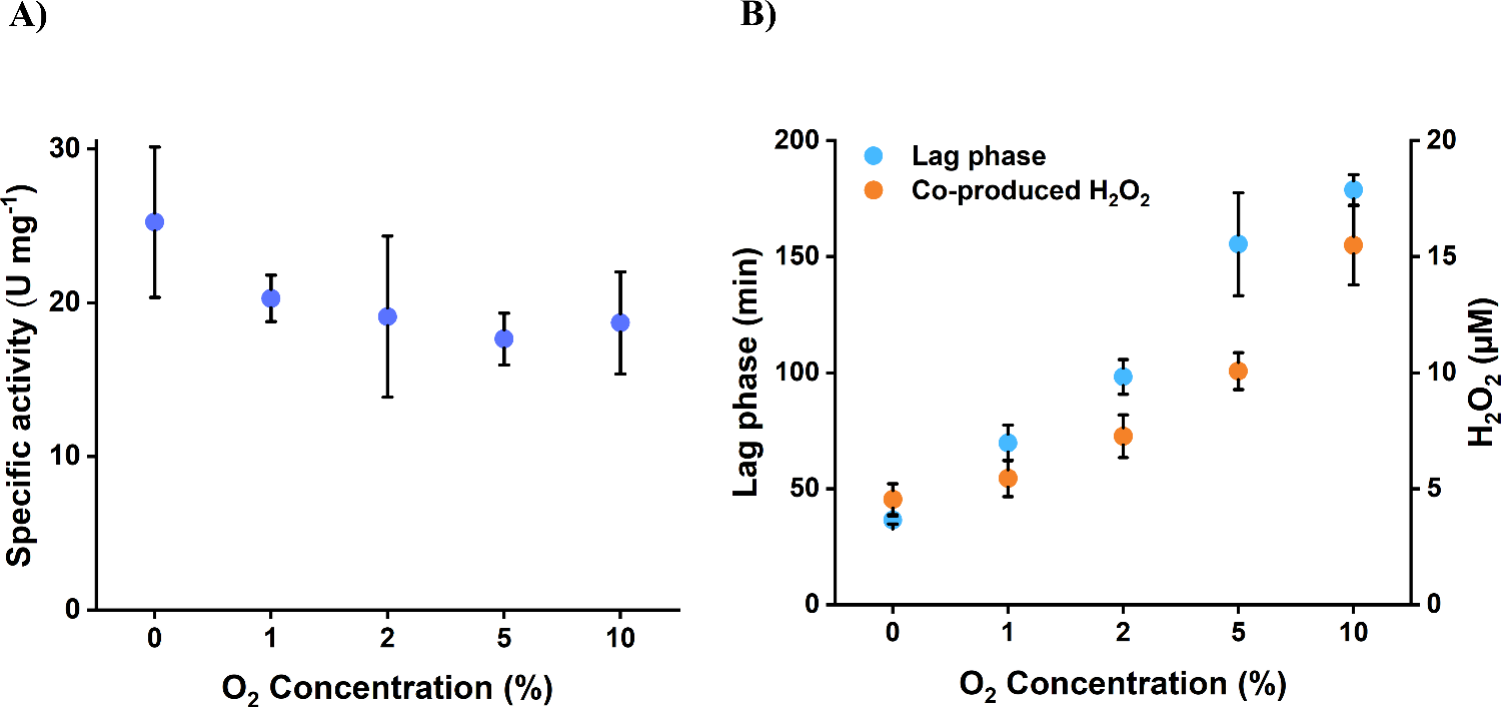
Investigation of the O_2_ effect on the activity of *Hp*SH (2 μg mL^–1^) (A), the length of lag phase, and hydrogen peroxide production (B). The measurements were performed at 30 °C using a reaction buffer containing 1 mM of NAD^+^, 50 mM Tris-HCl, 1 mM TCEP, and 1 μM FMN. The O_2_ concentrations used were adjusted by mixing H_2_- and O_2_-saturated reaction buffers according to the respective ratios. The reaction conditions used are further defined in the methods section.

On the other hand, the O_2_ content showed a significant (p-value of 1.06 x10^–6^) impact on prolonging the lag phase. A 10% O_2_ concentration resulted in a lag phase of up to 3 h (Figure 5B). We were unable to detect activities at 20% and 40% O_2_ concentrations. It is unclear whether this reflects the actual inability of the enzyme to scavenge O_2_ at high concentrations or an experimental artifact, possibly caused by the prolonged incubation at 30 °C, which may have affected the gas-tightness of the reaction vessel.

### Mechanism Insights

To gain further insights into the mechanism of O_2_ detoxification in *Hp*SH, we conducted a series of tests. First, we analyzed the concentration of H_2_O_2_ produced at the end of each lag phase (Figure 5B). This revealed a clear correlation between the initial O_2_ concentration and the H_2_O_2_ produced. This finding supports our hypothesis that, like *Cn*SH, *Hp*SH can detoxify O_2_ by reducing it into H_2_O_2_.^29^

More interestingly, incubation of *Hp*SH with catalytic amounts of NADH did not shorten the lag phase, as observed in *Cn*SH, but rather prolonged it (Table 1). The same trend was observed upon the addition of catalytic amounts of NADH and NADPH during activity assays, with NADPH causing a significantly more substantial effect (Table 1 and Figure S7). This suggests that both NADH and NADPH may have a regulatory effect on *Hp*SH, which differs from that of *Cn*SH.

**Table 1:** Effect of Catalytic Amounts of NAD­(P)­H on *Hp*SH Activity and Lag Phase^a^

	lag phase (min)	specific activity (U mg^–1^)
control, no NAD(P)H*	8 ± 3	26 ± 2.5
NADH	27 ± 2	26 ± 2
NADPH	38.5 ± 3	12.2 ± 1
NADH*	29.5 ± 3	27 ± 1

a *Hp*SH (2 μg mL^–1^) were directly added to a H_2_ -saturated pH 7 buffer containing 50 mM Tris, 1 mM NAD^+^, 1 mM TCEP, 1 μM FMN, and 1 μM NAD­(P)­H. For indicated samples with (*), *Hp*SH (5 μg mL^–1^) was preincubated on ice for 1 h with 1 μM of NADH before the measurement.

In addition, when analyzing the diaphorase activity in the absence of H_2_ using benzyl viologen, we observed a high activity of 11.7 U mg^–1^, without a lag phase, under anaerobic conditions (Figure S8). This indicates that the diaphorase domain of *Hp*SH functions independently of the hydrogenase module and its activity. Furthermore, we tested the reverse reaction—from NADH to H_2_—using a coupled system with xylose dehydrogenase (XDH) under anaerobic conditions as reported by Al-Shameri et al.^16^ Our previous experience with the system showed that a high concentration of NADH is required to drive the reaction toward H_2_; otherwise, the H_2_ level remains below the sensor’s detection limit. XDH can continuously supply NADH through the oxidation of xylose. In this setup, we observed H_2_ formation with a very short lag phase of approximately 30 s (Figure S9), indicating that electron transfer from NADH to the Ni–Fe center occurs rapidly in the absence of O_2_. It is important to note that 0.1 mM FMN is required for the XDH-hydrogenase coupled system. As expected, the yellow color of FMN turned colorless upon reduction. This reduced flavin can rapidly eliminate residual O_2_ *via* a rapid uncoupling reaction, thereby maintaining an O_2_-free environment.

To test whether NADH serves as the electron source for O_2_ detoxification, we conducted an H_2_-driven NAD^+^ reduction assay. After activating *Hp*SH, we introduced O_2_ and immediately observed a cessation of NADH formation (Figure S10). This was followed by a gradual decrease in the NADH signal, indicating its oxidation, presumably for the removal of O_2_. H_2_O_2_ could not be detected, as its concentration was below the detection limit of our assay. The anaerobic incubation of *Hp*SH with only H_2_ resulted in no activity, confirming that NADH, not H_2_, serves as the electron donor for eliminating residual putative O_2_ in *Hp*SH.

### Validity as an NADH Regeneration System

Finally, to underline the potential of *Hp*SH for cofactor regeneration in bioconversion, we coupled *Hp*SH with the commercial lactate dehydrogenase LDH to produce lactate from pyruvate. We used 8 mL sealed vessels filled with 1 mL buffer containing 1 mM NAD^+^ and 50 mM pyruvate, which was saturated with H_2_. The reaction was initiated by adding the enzymes (details in SI). Surprisingly, no detectable product formation occurred when both enzymes were added simultaneously. As the K_M_ value of LDH for NADH is lower than that of *Hp*SH for NAD^+^, the addition of LDH would cause an imbalance in the cofactor ratios, preventing *Hp*SH from self-activating. We decided to spectrophotometrically monitor the reaction *online* and add LDH after *Hp*SH activation (Figure S11). Analyzing the samples *via* HPLC revealed a high conversion of up to 75% after 6 h (Table S20). Thus, we were able to confirm the capability of *Hp*SH to be used as a cofactor regeneration system, with a TTN (μmol NADH regenerated: μmol *Hp*SH) of 2.23 × 10^5,^ comparable to *Cn*SH.^23^

## Discussion

The effects of NADH, along with the simultaneous addition of LDH and *Hp*SH during biotransformation, provided some insights into a potential mechanism underlying *Hp*SH reactivation.^29^ Unlike truly O_2_-tolerant hydrogenases like *Cn*MBH, which is characterized by having a high O_2_ tolerance factor (K_I_^O2, app^), *Hp*SH lacks such a feature. K_I_^O2, app^ is defined as the O_2_ concentration required to reduce H_2_ oxidation activity by 50%.^28^ This underscores that *Hp*SH must first remove residual O_2_ entirely before it can engage in cofactor reduction. Thereby making this enzyme more of an O_2_ scavenging enzyme, with O_2_ acting as a strong inhibitor, similar to the hydrogenase of cyanobacteria.^30,28^ Nevertheless, *Hp*SH demonstrated good O_2_ robustness, as only its lag phase was affected by elevating O_2_ concentrations, but not its activity, aligning with similar phenomena in other hydrogenases.^4^

The reactivation rate of *Hp*SH is O_2_-dependent and aligns with previous reports describing a lag phase caused by O_2_ in other hydrogenases. For example, *Cn*SH exhibits an O_2_-dependent lag phase; however, this phase is significantly shorter, approximately 1 min in duration.^31,29^ *Cn*SH can also be reactivated by NADH, NADPH, with or without the addition of H_2._ ^14^ The lag phase of the soluble hydrogenase from *Hydrogenophilus thermoluteolus* (*Ht*SH) is noticeably reduced or totally disappears by the addition of reducing agents or NADH.^32^ The [NiFe]-hydrogenase from *Allochromatium vinosum* is activated exclusively by H_2_, and its lag phase is independent of both enzyme and H_2_ concentrations, suggesting an intrinsic activation process.^33,34^ In contrast, *Hp*SH appears to require H_2_ and low amounts of self-produced NADH to be reactivated. *Hp*SH also displayed an autocatalytic reactivation behavior, as higher enzyme concentrations shorten the lag phase, a phenomenon also observed for other hydrogenases.^35^ Adding NADH and NADPH alone -even in catalytic amounts- appears to have a counter effect on *Hp*SH. A lag phase-dependent autocatalytic activation process, influenced by enzyme and cofactor concentration or the enzyme’s quaternary structure, has been reported for other hydrogenases.^35,36,12^

The rapid NADH oxidation activity of the diaphorase in the absence of H_2_, together with the immediate reverse reaction toward H_2_ production, indicates that reverse electron transfer is a fast process and that NADH serves as the electron source for O_2_ reduction.

The fact that *Hp*SH can still produce H_2_O_2_ and oxidize NADH upon O_2_ addition indicates a reductive activation mechanism through reverse electron transfer, like in *Cn*SH.^29^ Nevertheless, one must also consider that *Hp*SH is a flavoprotein and that H_2_O_2_ may be produced *via* a direct uncoupling reaction between the reduced flavin and O_2_. Yet the amount of H_2_O_2_ detected does not account for the total O_2_ in the system, indicating that O_2_ is also being reduced to something else, most likely water, as observed in other O_2_-tolerant hydrogenases.^9,28^ To confirm this, isotopically labeled O_2_ should be used, and the formation of isotopically labeled water must be verified. The functional characterization with LDH suggests an essential role of NADH in reactivation. Considering also the elongated lag phase observed after adding NADH, it appears that *Hp*SH reactivation operates in a sequential cooperative mechanism with H_2_ and NADH.

The following model illustrates the proposed reactivation mechanism (see Figure 6). Upon H_2_ binding, the small fraction of active *Hp*SH molecules, those without an O_2_ at the Ni–Fe center, slowly oxidize H_2_ and reduce NAD^+^, thereby producing small amounts of NADH, which are promptly released. The generated NADH can bind to the inactive *Hp*SH molecules, those bearing O_2_ at the Ni–Fe center, and, *via* reverse electron flow, reduce O_2_ (autocatalytic reactivation). O_2_ reduction occurs either at the Ni–Fe center or at the diaphorase module, where hydride transfer from NADH reduces the flavin, which subsequently transfers its electrons to O_2_ *via* rapid uncoupling.

**Figure 6:**
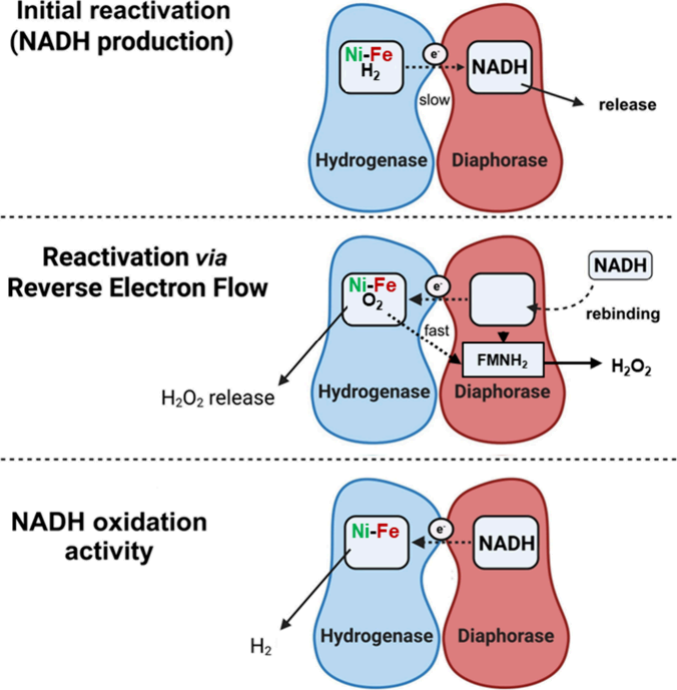
Proposed mechanism of the reactivation of *Hp*SH. The reactivation via reverse electron transfer is a cooperative process involving both NADH and H_2_.

With NADH alone, in the absence of O_2_, *Hp*SH acts as a proton-reducing enzyme and produces H_2_. This model explains why the simultaneous addition of LDH abolishes detectable product formation. LDH continuously withdraws the small amount of NADH produced by *Hp*SH, thereby maintaining the lag phase, as reactivation becomes solely dependent on H_2_. It also explains the rapid oxidation of NADH and H_2_ without a lag phase. The negative effect of NAD­(P)H in the presence of H_2_ cannot be explained at this stage. The simultaneous addition of both reductants, NADH and H_2_, might have caused a stagnation in the electron relay or changed the redox state of the Fe–S clusters. NAD­(P)H might also exhibit a regulatory phenomenon, such as negative cooperativity with H_2_, even at lower NAD­(P)­H concentrations. However, both interpretations remain highly speculative; spectroscopic and electrochemical analyses are necessary to validate these hypotheses.

Compared to *Cn*SH, *Hp*SH demonstrated better stability and a broader substrate spectrum (Table 2).

**Table 2:** Comparison of Protein Yield, Stability at 30 °C, and Substrate Spectrum of *Cn*SH and *Hp*SH

	*Hp*SH	*Cn*SH, reference
protein yield mg/g cells^a^	0.076	3.0–9.0,^29^
*k*_cat_(s^–1^)		
NAD^+^	137 ± 20	85 ± 3,^23^/690 ± 63,^25^
FMN	23 ± 7.3	20.3 ± 1.4,^26^
NADP^+^	2.1 ± 0.2	6.0 ± 0.4,^25^ ^b^
FAD	1.9 ± 0.4	0.49,^26^
K_M_ (mM)		
NAD^+^	0.4 ± 0.14	0.55 ± 0.08,^25^
FMN	n.d.	0.68 ± 0.11,^26^
NADP^+^	n.d.	0.60 ± 0.33,^25^ ^b^
FAD	n.d.	n.d.
half-life (h)	46.2	5.3^c^,^23^

a Homologue expression.

b NADP^+^ engineered variant.

c At 35 °C, 11 h at 25 °C. n.d. not determined.

Several issues still hinder the broad application of *Hp*SH in biotechnological applications. The primary challenge is its low protein yield—approximately 40 times lower than *Cn*SH—in its native host, combined with the slow growth rate of *H. pseudoflava*, which restricts its scalability. A potential solution would be to integrate the maturation machinery of *H. pseudoflava* into *E. coli* for heterologous expression. Notably, transferring *Cn*SH machinery into *E. coli* enabled the production of an active *Hp*SH in *E. coli*, but with significantly reduced activity compared to the native host.^37^ This discrepancy may not only result from an imbalance of maturation components, but it could also indicate that *Hp*SH maturation might follow a distinct pathway or involve active-site modifications. Spectroscopic evidence is needed to further investigate this possibility. Furthermore, unlike *Cn*SH, *Hp*SH exhibits a prolonged lag phase for O_2_ scavenging, which limits its use in aerobic setups. Nevertheless, its exceptional stability offers a valuable compromise for microaerobic and anaerobic applications, while providing easier handling compared to other, more O_2_-sensitive hydrogenases.^32,38^

## Conclusions

We successfully produced and purified *Hp*SH, an O_2_-scavenging soluble hydrogenase from *H. pseudoflava*. *Hp*SH catalyzed H_2_-driven NAD^+^ reduction with specific activity comparable to *Cn*SH, while exhibiting superior stability, retaining activity for over 72 h at 30 °C, and a half-life of 46 h. *Hp*SH also showed a better reduction activity of NADP^+^, FMN, and FAD compared to *Cn*SH. To our knowledge, this combination of cofactor flexibility, oxidative robustness, and long-lived activity has not been previously reported for any bidirectional hydrogenases. The enzyme’s delayed activation was linked to O_2_ levels, suggesting a detoxification mechanism similar to that of other hydrogenases. Notably, *Hp*SH required an H_2_ atmosphere and a specific NAD^+^/NADH ratio for activation. This highlights the need to further investigate its distinct activation mechanism and the nature of its active site.

## Materials and Methods

### Plasmid and Stain Generation

Since it was previously not possible to produce highly active *Hp*SH heterologously,^37^ the enzyme used in this study was produced within the native host. The recently identified genes coding for its four structural subunits, HoxFUYH, and the gene coding for the endopeptidase HoxW were expressed as an artificial operon. The pOCEx1 plasmid, used by Grenz et al., was employed.^18^ The individual genes were combined with artificial ribosome binding sites (RBS) and spacers generated by Siebert et al.^39^ and combined via Gibson assembly to create pOCEx1-StrepII-*hoxFUYH,* as is explained in detail in the supplements. Subsequently, the plasmid was transformed into and amplified in *E. coli* S17–1. To produce *Hp*SH, the previously produced plasmid was conjugated into *H. pseudoflava* DSM 1084 according to Grenz et al.,^18^ resulting in the production strain.

### Enzyme Expression and Purification

To produce *Hp*SH, the previously developed production strain was used to inoculate a TB-Agar plate with 25 μg mL^–1^ of Kanamycin. After 3 days of growth at 30 °C, a 5 mL preculture in LB media (10 g L^–1^ tryptone, 5 g L^–1^ yeast extract, 25 μg mL^–1^ of Kanamycin ) was inoculated from this plate, which was then grown at 30 °C and 120 rpm shaking for 8 h. This culture was subsequently used to inoculate. A second preculture of 50 mL in CO-Ox medium with 1% fructose, 25 μg mL^–1^ of Kanamycin, and 10 g MOPS L^–1^. This second preculture was then used to inoculate the main culture, which consisted of the same media with the addition of 1 mM IPTG. The CO-Ox medium used in this study followed the recipe specified by the DSMZ, which included 1% fructose and 10 g MOPS L^–1^, as per previous studies.^18^ Cells were grown until an optical density at 600 nm (OD_600_) of 4 was reached. Subsequently, the cells were harvested by centrifugation at 4 °C and 4,000 × g, washed in buffer (50 mM Tris-HCl and 150 mM KCl, pH 7.5), and frozen at −80 °C until further use. Before purification, the cells were thawed at 25 °C and lysed by sonication. The resulting lysate was separated by centrifugation (45000g, 4 °C, 1 h). The thereby generated soluble extract was purified using Strep-Tactin XT 4Flow high-capacity resin. The column was first equilibrated with buffer A (50 mM Tris-HCl, pH 8, containing 5% glycerol and 150 mM KCl). After applying the cell lysate, the column was washed with 5 CV buffer A with 5 mM NAD^+^. It was rewashed with 5 CV of the same buffer A; the bound protein was eluted with buffer A containing 50 mM biotin. The protein was concentrated using a Vivaspin column with a 50 kDa cutoff; subsequently, the elution buffer was exchanged with buffer A, and the protein was concentrated again.

### Enzyme Assays

To measure the H_2_-driven NAD^+^ reduction, ROTILABO glass cuvettes were filled with 2 mL of reaction buffer containing 50 mM Tris-HCl, 1 mM NAD^+^, 1 mM TCEP, and 1 μM FMN at a pH of 7. Subsequently, the cuvette was airtightly sealed and saturated with hydrogen for at least 15 min. This was conducted by bubbling the cuvette with pure H_2_ through a cannula while simultaneously allowing for a pressure exchange. Afterward, the reaction was started by adding purified enzyme via a gastight Hamilton syringe. The reaction was performed at 30 °C for at least 90 min and observed by measuring the absorption at 340 and 365 nm for NAD^+^ conversion.

The activity for NADP^+^ was determined under the same conditions with 1 mM NADP^+^ instead of NAD^+^. For Flavin-specific measurements, a buffer containing 50 mM Tris-HCl, 1 mM FAD/FMN, and 1 mM TCEP at pH 7 was used. The absorbance was monitored at 500 nm, using the protocol established by Al-Shameri et al.^26^ For the determination of the O_2_ effect on enzyme activity, a standard buffer was used at pH 8, containing 50 mM TRIS-HCl, 1 mM TCEP, and 1 μM FMN. The O_2_ concentration was adjusted by mixing aliquots of reaction buffer saturated with either H_2_ or O_2_. The cuvette was filled with the buffer to circumvent the formation of gas phase and thus explosive gas mixtures. Photometric measurements were performed at 365 nm for NAD­(P)H and at 500 nm for flavin-based cofactors, as previous studies have shown these wavelengths to be suitable for higher concentrations of cofactors.^26^

For H_2_O_2_ analysis, 200 μL sample out of the 2 mL cuvettes was taken at the end of NADH formation and measured spectrophotometrically in a plate assay at 498 nm using a coupled enzymatic assay of 5 U mL^–1^ Horseradish peroxidase with 0.5 mM 4-aminoantipyrin and 2 mM vanillic acid. Calibration was conducted using a defined H_2_O_2_ concentration. This assay was performed according to the method described by Al-Shameri et al.^26^

For the H_2_-independent NADH oxidation activity of *Hp*SH, a solution containing 50 mM Tris (pH 7.5), 2.5 mM benzyl viologen (BV), 0.5 mM NADH, and 1 mM dithionite was purged with argon for 20 min. Subsequently, 4 μg of *Hp*SH were added, and the absorbance at 600 nm was monitored. The same experiment was repeated without dithionite and argon purging. Negative controls were prepared identically, but without the enzyme. All experiments were conducted in independent biological triplicates.

For H_2_ production, a mixture containing 1 mL of 80 mM D-xylose, 1 mM NADH, and 100 μM FMN in 80 mM Tris-HCl (pH 7.5) was purged with argon. Excess amounts of XDH and lactonase were then added to drive the oxidation of D-xylose. Finally, 20 μg of *Hp*SH were introduced, and H_2_ production was monitored at room temperature without mixing using a Unisense H_2_ sensor as reported in Al-Shameri et al.^16^

### HPLC Analysis

The concentrations of pyruvate to lactate were measured by HPLC coupled with UV and RI detectors. Before HPLC analysis, samples were diluted in water (1:10), filtered with a spin filter, and then diluted (1:10) in 2.5 mM H_2_SO_4_. Ten μL were injected into the HPLC. The HPLC method followed the protocol published by Al-Shameri et al.^16^ The chromatograms were analyzed and integrated using CHROMELEON 6.80 SR15 software.

### Accession Codes

The enzyme characterized within this work consisted of the following Subunits (Table 3).

**Table 3:** Molecular Components of the Characterized Enzyme

subunit	accession code
HoxF	A0A4P6WYG5
HoxU	A0A4P6WVR5
HoxH	A0A4P6WSJ6
HoxY	A0A4P6WV23

## Supplementary Material



## Notes

Additional experimental information, genetic maps, protein sequences, and selected unprocessed data are listed in the supplement (PDF)
