# Supplementary material for: Biochemical and Mechanistic Insights into O2 Scavenging and Stability of the Soluble Hydrogenase from Hydrogenophaga pseudoflava
Source: ACS Omega. 2025 Dec 30;11(1):1769–77. doi: 10.1021/acsomega.5c09877 (PMC12809855; doi:10.1021/acsomega.5c09877)
Supplement: Supplementary file 1 [file ao5c09877_si_001.pdf]

Supplemental Information to:

Biochemical and Mechanistic Insights into O_2 Scavenging and Stability of the Soluble Hydrogenase from *Hydrogenophaga pseudoflava*

Dominik L. Siebert[a], Simon Grieshaber[b], Bastian Blombach[b], Volker Sieber[a,c], Ammar Al-Shameri[a,*]

[a)] Chair of Chemistry of Biogenic Resources, TUM Campus Straubing for Biotechnology and Sustainability, Technical University of Munich, Schulgasse 16, 94315, Straubing, Germany

[b)] Microbial Biotechnology, TUM Campus Straubing for Biotechnology and Sustainability, Technical University of Uferstraße 53, 94315, Straubing, Germany.

[c)] Catalytic Research Center, Technical University of Munich, 85748, Garching, Germany

[*] Corresponding author: a.al-shameri@tum.de

Table of Contents

Compositions of Carbon monoxide oxidizer medium- CO-Ox 4
Structural analysis and comparison of *Hp*SH 5
SDS-PAGE and UV-Vis spectrum utilised to evaluate *Hp*SH 6
Qualitative enzyme activity analysis by methylene blue reduction 7
Plasmid card of the used construct and construction method 8
Measured activities and lag phases at different pH, temperatures, and salts 9
Determination of substate spectrum 12
Determination of catalytic parameters Km and Kcat for NAD^+ 14
Investigation of O_2 effect on the lag phase and activity 15
Statistical significance of the analysed data sets. 15
Investigation of the NADH and NADPH influence on the Lag phase of *Hp*SH 17
The effect of O2 on the activity of HpSH after activation 17
H2-independent NADH activity of HpSH 18
H_2-production by *Hp*SH 20
Bioconversion of Pyruvate to Lactate using *Hp*SH 21
References 22
Appendix 23

List of abbreviations

**NAD^+/H:**	**nicotinamide adenine dinucleotide (oxidized/ reduced form)**
**H_2O_2**	**hydrogen peroxide**
**SDS-PAGE:**	**Sodium dodecyl sulphate polyacrylamide gel electrophoresis**
**PCR:**	**Polymerase chain reaction**
Hp:	***Hydrogenophaga pseudoflava***
Cn	***Cupriavidus necator***
**TTN**	**Total Turnover Number**

Compositions of Carbon monoxide oxidizer medium- CO-Ox

- Na_2HPO_4 x 12 H_2O 4.50 g
- KH_2PO_4 0.75 g
- NH_4Cl 1.50 g
- $MgSO_4$ x 7 H_2O 0.20 g
- $CaCl_2$ x 2 H_2O 0.03 g
- Ferric ammonium citrate 18.00 mg
- Distilled water 1000.00 ml
- Trace element solution SL-6 1.00 ml
- Trace element solution SL-6:
  - $ZnSO_4$ x 7 H_2O 0.10 g
  - $MnCl_2$ x 4 H_2O 0.03 g
  - H_3BO_3 0.30 g
  - $CoCl_2$ x 6 H_2O 0.20 g
  - $CuCl_2$ x 2 H_2O 0.01 g
  - $NiCl_2$ x 6 H_2O 0.02 g
  - Na_2MoO_4 x 2 H_2O 0.03 g
  - Distilled water 1000.00 ml

Structural analysis and comparison of *Hp*SH

As this work presents the first homologous expression of *Hp*SH, we performed a sequence analysis of this novel enzyme to identify its sequence similarity to *Cn*SH, possible binding sites, and conserved domains. This was done by aligning the individual subunits with their *Cn*SH counterparts, resulting in the similarities listed below. A complete sequence alignment of all subunits, as well as the endopeptidase HoxW, is added in the appendix.

Table S1. Ascension codes and amino acid similarity of HpSH compared to CnSH.

Subunit	Accession Code	Amino acid similarity to *Cn*SH [%]
HoxF	A0A4P6WYG5	41.9
HoxU	A0A4P6WVR5	33.9
HoxH	A0A4P6WSJ6	45.4
HoxY	A0A4P6WV23	45.8

In detail, we compared the individual subunits and their domains using both sequence and AlphaFold-based structures of the subunits, as listed on UniProt following the accession codes above. As the focus of this work is the biotechnological characterisation and application of *Hp*SH, we focused the structural comparison on potential cofactor binding sites and conserved proteolytic cleavage sites required for maturation. The resulting sites are summarised in Table S2. Firstly, we were able to identify a phosphate-binding loop in HpHoxF, like the one present in *Cn*SH, indicating a binding of NAD(P). Next, we searched for an FMN binding motif in HpHoxF and found a conserved region with over 80% similarity to the corresponding sequence in *CnHoxF*, implying a similar binding principle. For *Hp*HoxU and *Hp*HoxY, it was possible to identify potential iron sulphur clusters, as they are known to exist in CnSH. For *Hp*HoxH, it was possible to observe a similar binding pocket for the [Ni-Fe]-cofactor as previously observed for CnSH (Preisler).

Table S2. Comparison of potential cofactor binding sites in HpSH and CnSH

Subunit	Motive	CnSH		HpSH	
		Position	Sequence	Position	Sequence
HoxF	NAD(P) binding motif (GXGXXG)	219-224	GRGGAG	216-222	GRGGAG
	FMN binding motif (π–π stack)	332-379	GAGAYICGDES<br>ALIESCEGKRGTP<br>RVKPPFPVQQGY<br>LGKPTSVNNVET	331-378	GAGAY**V**CG**E**ES<br>ALIES**L**EG**Q**RGTP<br>R**IR**PPFPVQ**R**GY<br>LG**R**PT**V**VNNVET
	[4Fe–4S] binding site	C499-C502-C505-C545		C497-C500-C503-C547	
HoxU	[2Fe–2S] binding site	C35-C46-C49-C61		C34-C45-C48-C60	
	[4Fe–4S] binding site	C145-C148-C151-C198		C144-C147-C150-C198	
HoxY	[4Fe–4S] binding site	C41-C44 -C113-C179		C20-C23- -C91-C146	
HoxH	[Ni-Fe-]-active site	C65-P390-R391-C461		C80-P392-R393-C462	

As the exact mechanism by which endopeptidase HoxW maturates HoxH has not been precisely investigated, it was not possible to determine a specific restriction site for this process. However, it is known that the cut fragment in *C. necator* has a length of 24 amino acids, leading to the assumption that HoxW cuts after a histidine residue, as it has been identified for other hydrogenase maturation systems. Following this assumption, we performed a multiple sequence alignment of the hydrogenases recently expressed using our universal expression platform (DLS) and observed a conserved THALG region surrounding the expected cutting site of *HoxW*. This might indicate *that HpHoxW cuts in a similar region, resulting in the cleavage of 43 amino acids in HpSH, which would lead to a reduction in molecular mass of approximately 4.6 kDa for the mature* protein.

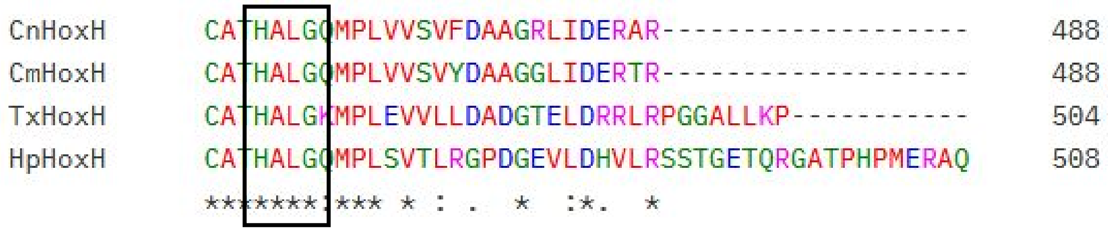

SDS-PAGE and UV-Vis spectrum utilised to evaluate *Hp*SH

To determine the composition and purity of *Hp*SH, a 15% SDS-PAGE was performed. Interestingly, it shows a clear four-subunit pattern. Yet the observed bands of *HoxH* and *HoxU* did not resemble the expected sizes, even considering the predicted size of HoxH after maturation by *HoxW*. Therefore, the slightly altered flow conditions may be due to their individual amino acid compositions, as the structure of the particular protein can also influence SDS-PAGE results.

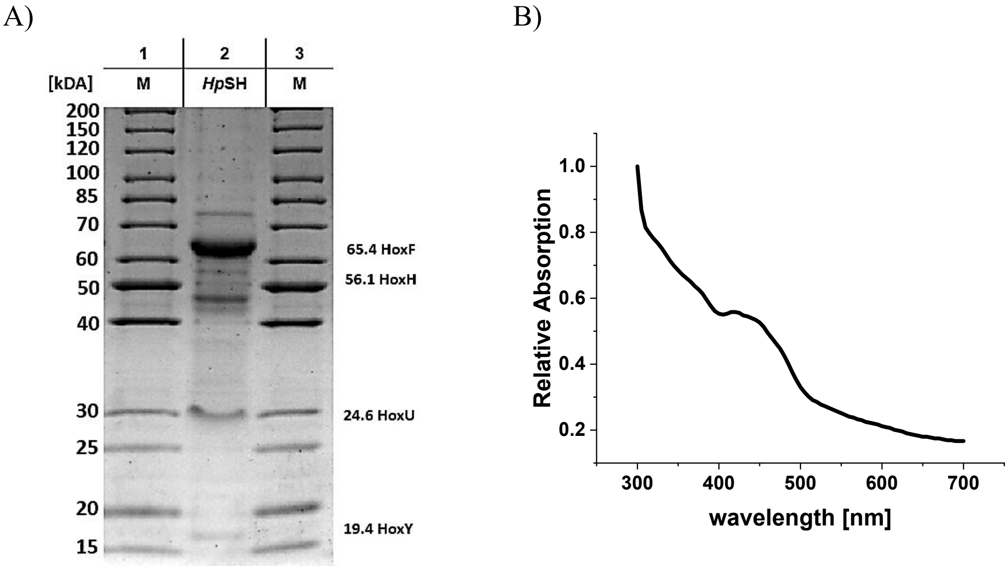

Figure S1. A) SDS-PAGE prepared to investigate the purity and subunit composition of *Hp*SH, B) UV-Vis spectrum showing a peak between 400 and 500 nm similar to CnSH *(1)*

Qualitative enzyme activity analysis by methylene blue reduction

Since no activity for the H_2-driven NAD^+ reduction could be detected, a qualitative long-term assay was performed to evaluate whether the enzyme is generally inactive. For this, 2 mL of buffer containing 50 mM TRIS-HCl, 0.1 mM methylene blue, and one µM FMN at pH eight was added to an airtight glass vial, sealed, and saturated with hydrogen. Subsequently, *Hp*SH was added, except for negative controls. Subsequently, the vials were incubated for 8 hours in the dark, resulting in a destaining of the enzyme-containing vials, implying a general reductive activity of the enzyme. Although the negative control was lighter than expected, a clear colour difference could be observed.

A)

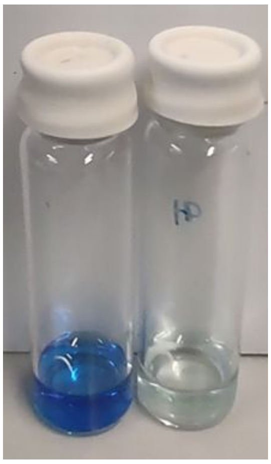

B)

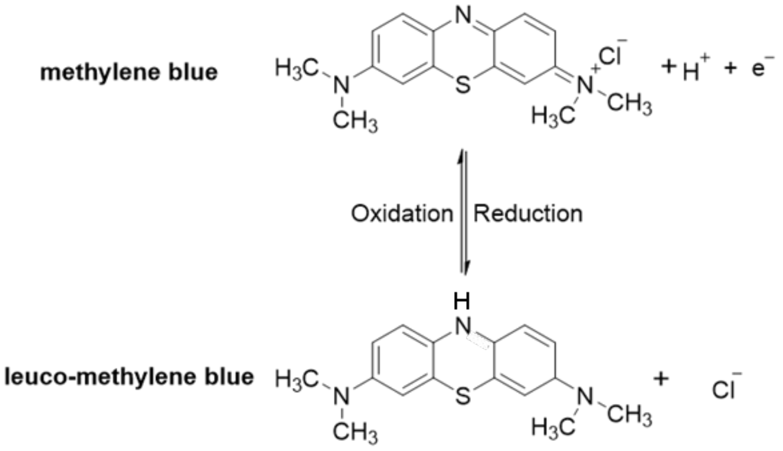

Figure S2 A) Vials showing the qualitative activity of *Hp*SH by visualizing the hydrogen-induced reduction of methylene blue, the left vial showing a negative control, while the right vial showed the sample. B) as well as the performed assay reaction.

Plasmid card of the used construct and construction method

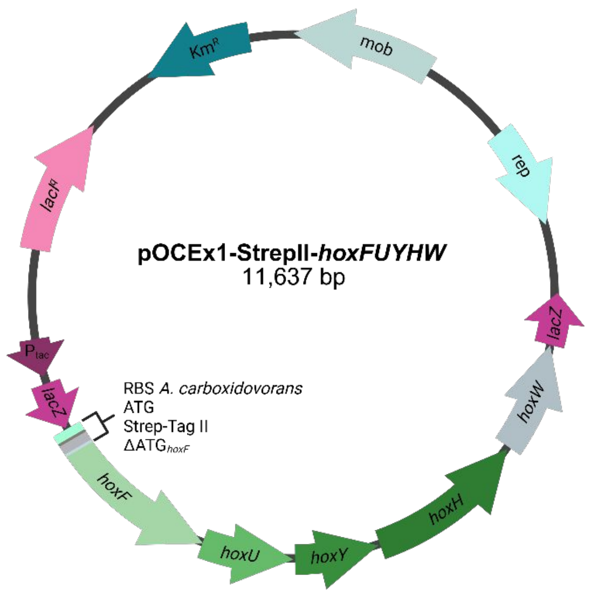

Figure S3. Plasmid map of the artificial operon containing the structural genes *HoxFUYH* used to overproduce *Hp*SH, as well as the endopeptidase *HoxW*. The genes are complemented with an artificial RBS based on *Afipia carboxidovorans* and a strep TagII.

To construct the used plasmid, the POCEx1 vector, previously used by Grenz et al., was employed. *(2)*. was linearised with BamHI. Subsequently, the structural genes *HoxFUYH*, as well as the endopeptidase HoxW, were amplified via PCR using the primers listed below and integrated via isothermal recombination via Gibson. *(3)*. Alongside the genes, additional ribosomal binding sites from *A. carboxidovorans* as well as a spacer sequence (5'-GAGGAGAACGGA-3') *(4)* and a *Strep*-TagII *(5)* were added. The final plasmid was integrated into chemically competent *E. coli* S17-1 cells. Subsequently, the plasmid was verified by Oxford Nanopore sequencing by Microsynth Seqlab GmbH (Göttingen, Germany).

Used Primers:

Forward:
TTCCTGCAGCCCGGGGGATCGAGGAGAACGGAATGGCTAGCTGGAGCCACCCGCAGTTC
GAAAAAGGCGCCACCGCCTTTGACCAGCC

Reverse:
CGGCCGCTCTAGAACTAGTGTCATGCGGGGACCTCTTCC

.

Measured activities and lag phases at different pH, temperatures, and salts

All measurements in this work were performed in biological triplicates grown and harvested according to the methods stated in the main article. The purification of Strep-tagged enzymes was performed via Strep-TactinXT columns according to the public IBA protocol. Concentration measurements were performed via Bradford. The experiments resulting in the data presented in Tables S1-S8 were performed using ROTILABO glass cuvettes filled with 2 mL of reaction buffer containing 50 mM Tris-HCl, 1 mM NAD^+, 1 mM TCEP, and 1 µM FMN at pH 8 and 30 °C. For temperature optimization, the pH was modified to counter the temperature dependence of Tris. For pH optimisation, 50 mM of MES was used for samples with a pH below 7.

Table S1. Hydrogen-induced activities on NAD^+ of the individual purifications of *Hp*SH at different temperatures, and the resulting mean and standard deviation

Temperature [°C]	Purification 1 [U/mg]	Purification 2 [U/mg]	Purification 3 [U/mg]	Average [U/mg]	Deviation [U/mg]
**20**	1.61	1.24	0.85	1.24	0.18
**30**	20.54	30.32	24.86	25.24	4
**40**	17.5	20.76	22.4	20.22	2.03
**50**	0.07	0.08	0.07	0.07	0.01
**60**	0.06	0.08	0.11	0.08	0.02

Table S2 Lag phases of the individual purifications of *Hp*SH at different temperatures and the resulting mean and deviation

Temperature [°C]	Purification 1 [min]	Purification 2 [min]	Purification 3 [min]	Average [min]	Deviation [min]
**20**	80	86	>90*	82	3
**30**	38	38	34	37	2
**40**	52	38	51	47.	6
**50**	-	-	-	-	-
**60**	-	-	-	-	-

* The lag phase ended around 90 minutes, which interfered with the end of the measurement and thereby was imprecise.

Table S3. Hydrogen-induced activities on NAD^+ of the individual purifications of *Hp*SH in the presence of the stated concentration of KCl, and the resulting mean and standard deviation

KCl conc. [mM]	Purification 1 [U/mg]	Purification 2 [U/mg]	Purification 3 [U/mg]	Average [U/mg]	Deviation [U/mg]
**0**	20.54	30.32	24.86	25.24	4
**50**	17.7	25.18	29.47	24.12	4.86
**100**	16.26	24.01	16.76	19.01	3.54
**150**	10.92	15.11	12.69	12.91	1.72
**200**	9.38	8.43	16.38	11.4	3.55
**250**	7	9.86	11.94	9.6	2.03

Table S4 Lag phases of the individual purifications of *Hp*SH in the presence of the stated concentration of KCl and the resulting mean and deviation

KCl conc. [mM]	Purification 1 [min]	Purification 2 [min]	Purification 3 [min]	Average [min]	Deviation [min]
**0**	38	38	34	37	2
**50**	39	44	15	33	13
**100**	46	41	20	36	11
**150**	59	64	26	50	17
**200**	60	71	28	53	18
**250**	68	40	35	48	15

Table S5. Hydrogen-induced activities on NAD^+ of the individual purifications of *Hp*SH in the presence of the stated concentration of NaCl, and the resulting mean and standard deviation

NaCl conc. [mM]	Purification 1 [U/mg]	Purification 2 [U/mg]	Purification 3 [U/mg]	Average [U/mg]	Deviation [U/mg]
**0**	20.54	30.32	24.86	25.24	4
**50**	18.86	18.03	21.76	19.55	1.6
**100**	16.56	14.56	21.76	17.63	3.03
**150**	9.94	15.29	17.47	14.23	3.16
**200**	11.9	7.27	14.8	11.32	3.1
**250**	4.18	7.93	10.26	7.46	2.5

Table S6 Lag phases of the individual purifications of *Hp*SH in the presence of the stated concentration of NaCl and the resulting mean and deviation

NaCl conc. [mM]	Purification 1 [min]	Purification 2 [min]	Purification 3 [min]	Average [min]	Deviation [min]
**0**	38	38	34	37	2
**50**	47	52	33	44	8
**100**	49	25	38	37	10
**150**	65	20	52	46	19
**200**	28	60	22	37	17
**250**	64	47	43	51	9

Table S7. Hydrogen-induced activities on NAD^+ of the individual purifications of *Hp*SH at different pH values, and the resulting mean and standard deviation

pH [-]	Purification 1 [U/mg]	Purification 2 [U/mg]	Purification 3 [U/mg]	Average [U/mg]	Deviation [U/mg]
**5.5**	8.23	10.08	10.82	9.71	1.09
**6**	31.65	30.27	13.26	25.06	8.36
**6.5**	33.65	29.15	23.09	28.63	4.33
**7**	34.14	35.34	32.01	33.83	1.38
**7.5**	24.49	27.93	27.29	26.57	1.49
**8**	20.54	30.32	24.86	25.24	4
**8.5**	8.32	4.68	8.94	7.32	1.88
**9**	0.26	0.26	0.26	0.26	0

Table S8 Lag phases of the individual purifications of *Hp*SH at different pH values and the resulting mean and deviation

pH [-]	Purification 1 [min]	Purification 2 [min]	Purification 3 [min]	Average [min]	Deviation [min]
**5.5**	19	17	16	17	1
**6**	24	15	25	21	4
**6.5**	18	25	25	23	3
**7**	16	31	22	23	6
**7.5**	26	25	50	34	12
**8**	38	38	34	37	2
**8.5**	66	77	71	71	4
**9**	-	-	-	-	-

Determination of substate spectrum

The experiments yielding the data presented in Tables S9 and S10 were conducted using ROTILABO® glass cuvettes filled with 2 mL of reaction buffer containing 50 mM Tris-HCl, 1 mM cofactor, 1 mM TCEP, and 1 µM FMN at pH 7 and 30 °C. For Flavin cofactors, no additional FMN was added.

Table S9. Hydrogen-induced activities on 1 mM of different cofactors of the individual purifications of *Hp*SH and the resulting mean and standard deviation

Cofactor	Purification 1 [U/mg]	Purification 2 [U/mg]	Purification 3 [U/mg]	Average[U/mg]	Deviation [U/mg]
**$NADP^+$**	0.70	0.86	0.78	0.78	0.07
**FAD**	2.17	2.16	1.31	1.88	0.4
**FMN**	8.35	5.34	11.82	8.5	2.65

Table S10. Lag phases were measured on 1 mM of different cofactors in the individual purifications of *Hp*SH, and the resulting mean and deviation

Cofactor	Purification 1 [min]	Purification 2 [min]	Purification 3 [min]	Average [min]	Deviation [min]
**$NADP^+$**	22	26	32	26	5
**FAD**	98	95	144	112	22
**FMN**	79	85	86	83	3

To test whether the low conversion observed for NADP+ could be an artifact, a conversion was performed using more HpSH, resulting in a final concentration of 40 µg/ml in the cuvette. This led to a shortened lag phase compared to the experiments with 2 µg/ml of enzyme. For FAD and FMN, to ensure that the observed conversion is indeed caused by the enzyme and not by photodegradation, negative controls were performed for all flavin conversions containing identical buffers and hydrogen, but no enzyme.

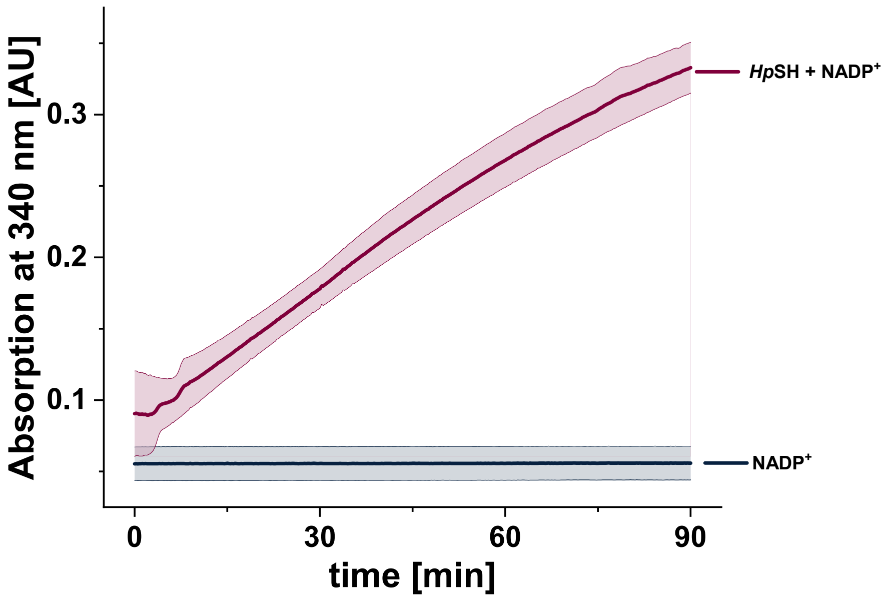

Figure S4. Conversion of NADP+ by *Hp*SH, in contrast to negative controls containing identical buffer substances and hydrogen, but no *Hp*SH.

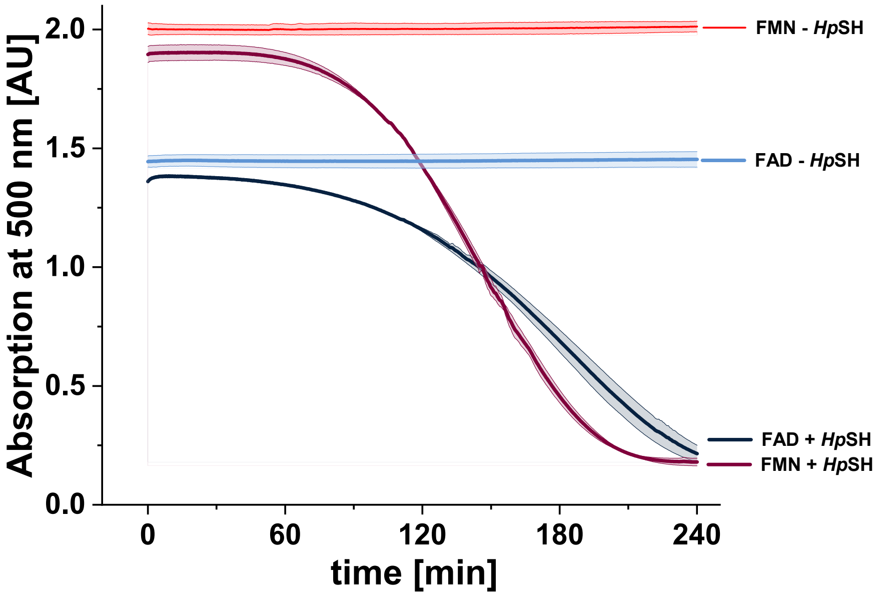

Figure S5. Conversion of flavin cofactors by *Hp*SH, in contrast to negative controls containing identical buffer substances and hydrogen, but no *Hp*SH.

Determination of catalytic parameters Km and Kcat for NAD^+

The experiments resulting in the data presented in Table S11 were performed using ROTILABO glass cuvettes filled with 2 mL of reaction buffer containing 50 mM Tris-HCl, 25-1000 µM NAD^+

, 1 mM TCEP, and 1 µM FMN at pH 7 and 30 °C.

Table S11. Hydrogen-induced activities on NAD^+ of the individual purifications of *Hp*SH at different NAD^+ concentrations, and the resulting mean and standard deviation

NAD [mM]	Purification 1 [U/mg]	Purification 2 [U/mg]	Purification 3 [U/mg]	Average[U/mg]	Deviation [U/mg]
**25**	2.66	1.38	1.53	1.86	0.57
**50**	3.69	3.26	3.42	3.46	0.17
**125**	8.4	7.93	8.32	8.22	0.2
**250**	22.71	24.36	17.69	21.59	0.83
**500**	32.09	29.01	29.38	30.16	1.37
**1000**	34.15	35.35	31.99	33.83	1.39

From this data, a plot was created, which was subsequently used to determine the parameters Km, Kcat, and Km/Kcat according to the Michaelis-Menten formula. Calculations were performed by modeling each triplicate individually via its origin, with a fixed v_{max} value.

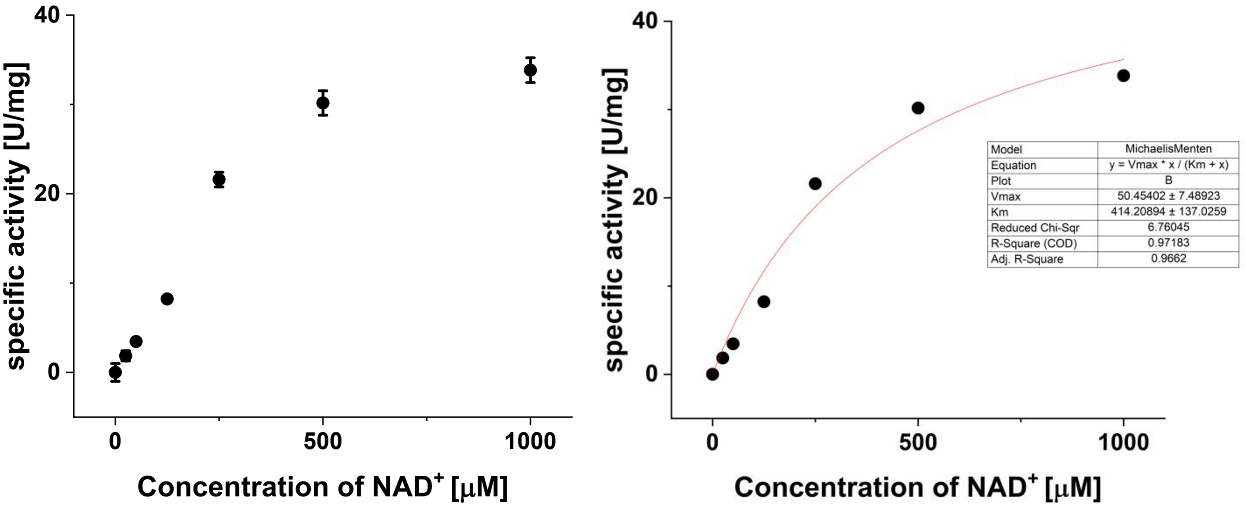

Figure S6. Plot of the specific activity of *Hp*SH against the NAD^+ concentration (left). The Michaelis-Menten fit with the corresponding values (right).

Investigation of the O_2 effect on the lag phase and activity

The experiments resulting in the data presented in Table S12-4 were performed using ROTILABO glass cuvettes filled with 2 mL of reaction buffer containing 50 mM Tris-HCl, 25-1000 µM NAD^+, 1 mM TCEP, and 1 µM FMN at pH 8 and 30 °C.

Table S12. Hydrogen-induced activities on NAD^+ of the individual purifications of *Hp*SH in the presence of different oxygen concentrations, and the resulting mean and standard deviation

Oxygen ratio [%]	Purification 1 [U/mg]	Purification 2 [U/mg]	Purification 3 [U/mg]	Average [U/mg]	Deviation [U/mg]
**0**	20.55	30.33	24.85	25.24	4
**1**	18.39	22.12	20.31	20.27	1.52
**2**	26.47	15.59	15.2	19.09	5.23
**5**	19.55	15.43	17.93	17.64	1.69
**10**	22.61	14.5	18.93	18.68	3.32

Table S13: Lag phases of the individual purifications of *Hp*SH in the presence of different oxygen concentrations and the resulting mean and deviation

Oxygen ratio [%]	Purification 1 [min]	Purification 2 [min]	Purification 3 [min]	Average [min]	Deviation [min]
**0**	38	38	34	37	2
**1**	81	63	66	70	8
**2**	88	103	104	98	7
**5**	131	185	151	156	22
**10**	170	186	180	179	7

Table S14: Amount of H_2O_2 coproduced by individual purifications of *Hp*SH in the presence of different oxygen concentrations and the resulting mean and deviation

Oxygen ratio [%]	Purification 1 [mM]	Purification 2 [mM]	Purification 3 [mM]	Average [mM]	Deviation [mM]
**0**	5.24	4.74	3.64	4.54	0.67
**1**	6.5	4.65	5.16	5.44	0.78
**2**	7.6	8.19	6	7.26	0.92
**5**	10.55	10.71	8.95	10.07	0.8
**10**	16.1	17.2	13.16	15.49	1.71

Statistical significance of the analysed data sets.

To evaluate the statistical significance of the produced datasets, they were analyzed via Origin by creating an ANOVA test, which determined the p-values of the observed variations. This was done to evaluate the significance of different O_2 concentrations towards the activity and lag phase observed for *Hp*SH. This resulted in the following parameters.

Table S15: F- and P values calculated for the O_2-dependent activity and lag phase generation

Dataset	F value	p-value
**O_2-depending activity**	1.49	0.27
**O_2-dependent lag phase**	53.05	$1.06 \cdot 10^{-6}$

Since a statistical significance can be assumed if the p-value is < 0.05, there is an implied strong statistical significance for the O_2-dependent lag phase creation, but none for the changes in activity.

Investigation of the NADH and NADPH influence on the Lag phase of *Hp*SH

To test the influence of NADH preincubation on *Hp*SH, we prepared a stock solution containing 0.5 mg/ mL of the enzyme. Half of this stock solution was then incubated on ice for 1 hour in a pH 7 buffer containing 50 mM Tris and 1 µM NADH. The other half used as a control was incubated in the same way without adding NADH. Both samples were then tested for their activity and the length of their lag phase using ROTILABO® glass cuvettes filled with 2 mL of reaction buffer containing 50 mM Tris-HCl, 1 mM µM NAD^+, 1 mM TCEP, and 1 µM FMN at pH 7 and 30 °C.

Table S16. Length of the lag phase with and without preincubation with 1 µM NADH

	**Purification 1 [min]**	**Purification 2 [min]**	**Purification 3 [min]**	**Average [min]**	**Deviation [min]**
**- NADH**	3.00	10.01	10.26	7.76	3.36
**+ NADH**	27.51	26.76	34.26	29.51	3.37

To test the influence of NADH and NADPH on *Hp*SH, we prepared a pH 7 buffer containing 50 mM Tris, 1 mM NAD^+, 1 mM TCEP, 1 µM FMN, and 2 µg/ml enzyme. To test the effect of NAD(P)H on the activity and the lag phase of *Hp*SH, we added 1 µM of the corresponding cofactor to the respective samples and monitored the activity. Interestingly, this resulted in a delayed lag phase for the addition of NADH and even a reduced activity for NADPH.

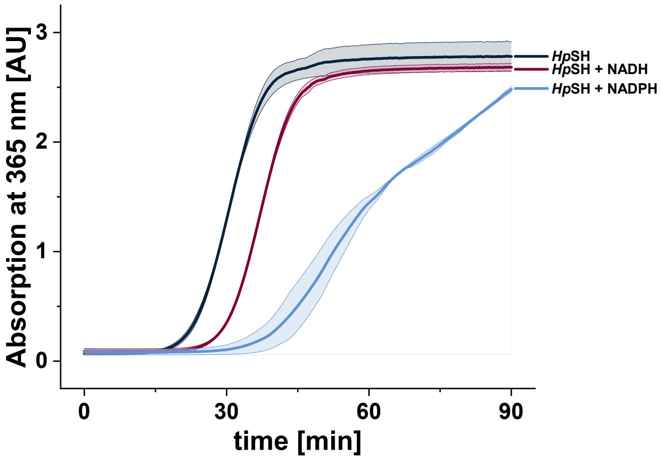

Figure S7. Photometric measurement of the NAD^+ conversion of *Hp*SH in the presence of NADH and NADPH

Table S17. The activity of *Hp*SH with and without preincubation with 1 µM NAD(P)H

**Specific activity [U/mg]**	**Purification 1 [U/mg]**	**Purification 2 [U/mg]**	**Purification 3 [U/mg]**	**Average [U/mg]**	**Deviation [U/mg]**
**Control**	29.67	25.38	23.62	26.22	2.54
**+ NADH**	28.42	26.84	26.66	27.31	0.79
**+NADPH**	10.33	13.32	12.81	12.15	1.31

The effect of O_2 on the activity of *Hp*SH after activation

To test the influence of O_2 on *Hp*SH after activation. 2mL of Tris-HCl, pH 7.0, with 1 mM NAD^+, 1 mM TCEP, and 1 µM were purged with H_2. Finally, 20 µg of HpSH were added to initiate the reaction at 30°C. Air was injected into the liquid phase either directly after the activation of *Hp*SH started with O_2 final conc. 5% (equals 500 µL air), or at the end of NADH formation with O_2 final conc. 1% (equals 100 µL air). O_2 is clearly an inhibitor of the HpSH; it also shows that scavenging of O_2 is permitted through NADH oxidation.

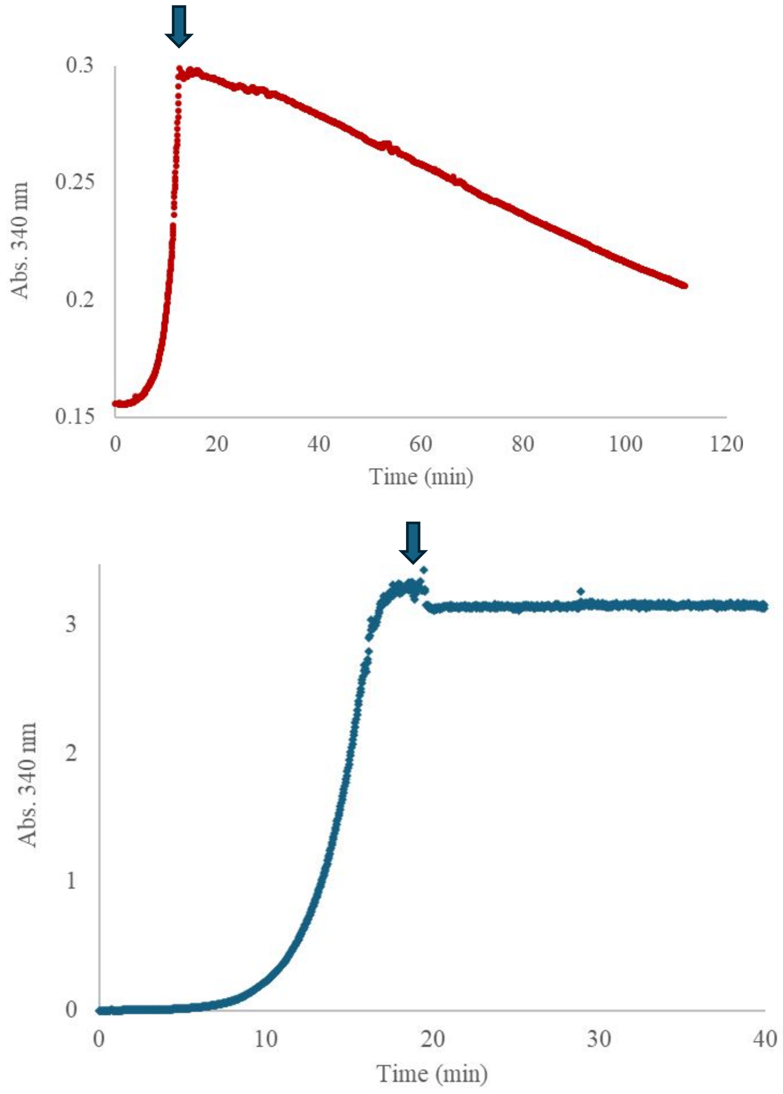

Figure S8. Photometric measurement of the NAD^+ conversion by *Hp*SH. Arrows indicate the air injection time point. 500 µL of air was injected directly after activation (upper graph) or after NADH formation with 100 µL of air (lower graph)

H_2-independent NADH activity of *Hp*SH

50 mM Tris pH 7.5, 2.5 mM of benzyl viologen (BV), 0.5 mM NADH, and 1 mM dithionite were purged with Ar for 20 min. Then 4 µg of *Hp*SH were added, and the absorption at 600 nm was monitored. The negative control was prepared the same way, but without the enzyme. A rapid increase in the reduction of BV was observed in the samples with the enzyme, indicating H_2-independent diaphorase activity of 11.7 U mg^{-1}. The same was tested without the addition of dithionite and purging with Ar, still we observed an increase in the single at 600 nm, but with much less intensity.

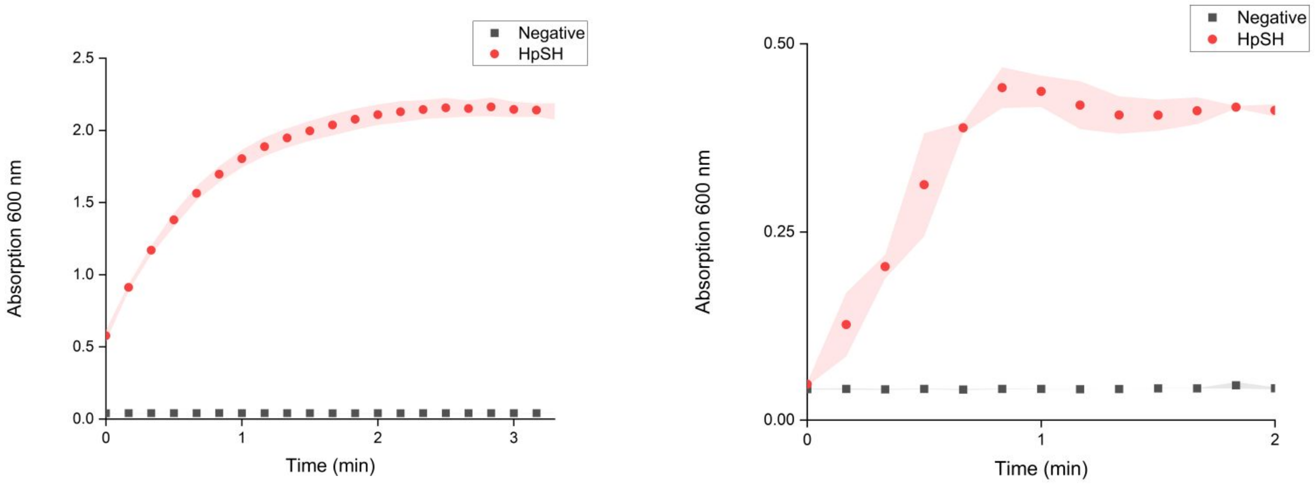

Figure S9: NADH oxidation activity of *Hp*SH on BV without H_2. Under anaerobic conditions (left) and at atmospheric conditions without addition of dithionite or purging with argon (right)

H_2-production by *Hp*SH

To test H_2 production, *Hp*SH, a coupled reaction involving xylose dehydrogenase (XDH) and lactonase, was carried out similarly to Al-Shameri *et al. (6)*. In short, the XDH will oxidize D-xylose and reduce NAD^+, which will be taken and oxidized by *Hp*SH to produce H_2.

1 mL of 80 mM D-xylose and 1 mM NADH, along with 100 µM FMN in 80 mM Tris-HCl, pH 7.5, were purged with argon. Then, excessive amounts of XDH and lactonase were added to drive the D-xylose oxidation. Finally, 20 µg of *Hp*SH were added, and the H_2

production was monitored at room temperature without mixing using a Unisense H_2 sensor similar to Al-Shameri *et al. (6)*

The production of H_2 by *Hp*SH starts immediately after adding the enzyme at a rate of up to 0.56 nmol min^{-1}.

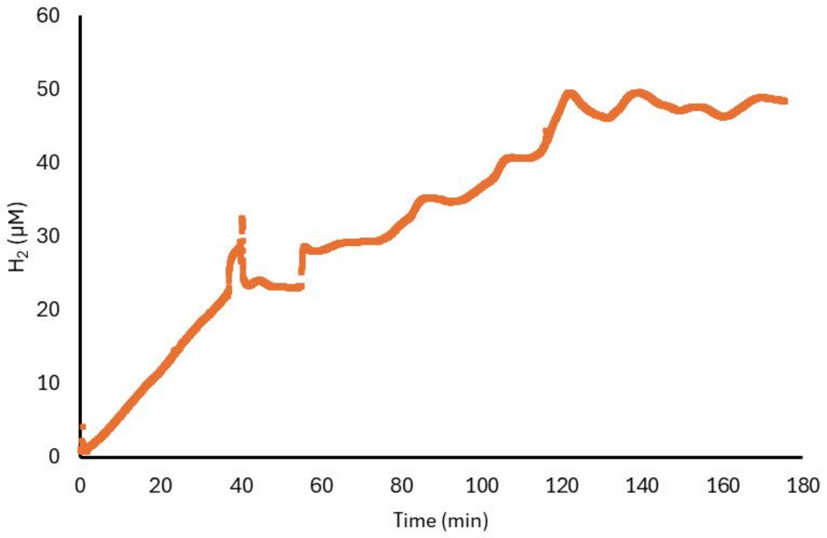

Figure S10: The production of H_2 *Hp*SH during the oxidation of D-xylose by XDH.

Bioconversion of Pyruvate to Lactate using *Hp*SH

To prove the capability of *Hp*SH to be applied as a cofactor regeneration system in bioconversion, we used commercial LDH (1.1.1.27) from Carl ROTH in ROTILABO® glass cuvettes filled with 2 mL of reaction buffer containing 100 mM Tris HCl, 50 mM pyruvate, 1 mM NAD^+, 1 mM TCEP, 1 µM FMN at pH 7. First, we attempted to apply it as a one-pot system, saturating the system with H_2 and subsequently adding 2 U of *Hp*SH and 6 U of LDH via a gastight syringe. This resulted in the conversion and TTN listed below after 12 hours of incubation.

Table S18. Lactate is produced in *the HpSH-coupled* LDH one-pot system with simultaneous enzyme addition.

	Purification 1 [mM]	Purification 2 [mM]	Purification 3 [mM]	Average [mM]	Deviation [mM]
***Hp*SH**	Not detectable	Not detectable	Not detectable	Not detectable	Not calculatable

To investigate whether the simultaneous addition of LDH caused the absence of activity for *Hp*SH, we preincubated HpSH and then added LDH, following the same method described above. For this, we performed an online observation of the NADH generation and utilization by measuring the UV at 365 nm for 1 hour.

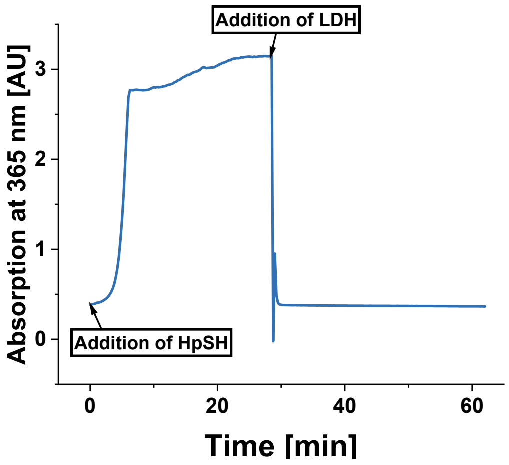

Figure S11. Photometric online observation of the NADH generation and consumption by *Hp*SH and LDH

Afterwards, we continued the reaction for another 5 hours, and analysed them *via* HPLC, resulting in the amounts of lactate and the calculated TTN values listed below.

Table S19. Lactate is produced in *the HpSH-coupled* LDH one-pot system with individual enzyme addition.

	Purification 1 [mM]	Purification 2 [mM]	Purification 3 [mM]	Average [mM]	Deviation [mM]
***Hp*SH**	40.31 0	36.81	36.65	37.92	1.69

Table S20. TTN of *Hp*SH (µmol of lactate: µmol *Hp*SH)

	Purification 1	Purification 2	Purification 3	Average	Deviation
***Hp*SH**	$2.37 \cdot 10^5$	$2.16 \cdot 10^5$	$2.15 \cdot 10^5$	$2.23 \cdot 10^5$	$9.92 \cdot 10^4$

References

1. Schiffels, J., Pinkenburg, O., Schelden, M., Aboulnaga, E.-H. A. A., Baumann, M. E. M., and Selmer, T. (2013). An innovative cloning platform enables the large-scale production and maturation of an oxygen-tolerant NiFe-hydrogenase from Cupriavidus necator in Escherichia coli, as reported in *PLOS ONE 8,* e68812.

2. Grenz, S., Baumann, P. T., Rückert, C., Nebel, B. A., Siebert, D., Schwentner, A., Eikmanns, B. J., Hauer, B., Kalinowski, J., Takors, R., and Blombach, B. (2019). Exploiting Hydrogenophaga pseudoflava for aerobic syngas-based production of chemicals, *Metabolic engineering 55,* 220–230.

3. Gibson, D. G., Young, L., Chuang, R.-Y., Venter, J. C., Hutchison, C. A., and Smith, H. O. (2009) Enzymatic assembly of DNA molecules up to several hundred kilobases, *Nature methods 6,* 343–345.

4. Siebert, D., Busche, T., Metz, A. Y., Smaili, M., Queck, B. A. W., Kalinowski, J., and Eikmanns, B. J. (2020) Genetic Engineering of Oligotropha carboxidovorans Strain OM5-A Promising Candidate for the Aerobic Utilization of Synthesis Gas, *ACS Synthetic Biology 9,* 1426–1440.

5. Schmidt, T. G. M., and Skerra, A. (2007). The Strep-tag system for one-step purification and high-affinity detection or capturing of proteins, Nature Protocols *2,* 1528–1535.

6. Al-Shameri, A., Siebert, D. L., Sutiono, S., Lauterbach, L., and Sieber, V. (2023) Hydrogenase-based oxidative biocatalysis without oxygen, *Nature communications 14,* 2693.

Appendix

HoxF Alignement

```
CnHoxF      --------MDSRITTILERYRSDRTRLIDILWDVQHEYGHIPDAVLPQLGAGLKLSPLDI   52

CmHoxF      --------MSKDIRTILERYRSDRARLMDILWDVQHLYGHIPDEVLPQLAAELNLSPLDI   52

TxHoxF      MQTTVTPDPAGPIPAVLARHGHRPHRLLQILHDVQDLYGWLPPEALTAVAHGLHLPRVAV   60

HpHoxF      MTAFDQPSALHAADAVLARWQHDPHALVQVLRETQAQTHWLPRELLAHIAAALRLTTAHV   60

                          ::* *       *:::* :.*     :*   *  :.  *.*    :

CnHoxF      RETASFYHFFLDKPSGKYRIYLCNSVIAKINGYQAVREALERETGIRFGETDPNGMFGLF   112

CmHoxF      RETASFYHLFHDKPSGKHRIYLCNSVIAKMNGYQAVHDALERETGVRFGETDPNGMFGLF   112

TxHoxF      ESTAGFYSFLYTRPVGQYRVLFSDNITDCLLGAPALMQRLCERLWLEPGKVSPDGLASVA   120

HpHoxF      EGVASFYRFFHLRPVGRVHVLFSDNITDRMAGSDALMARLCARLGVAPGQVDAQGRFCVD   120

            . .*.** ::  :* *: :: :.:.:   : *  *:   *  .  :  *:.. :*   :

CnHoxF      DTPCIGLSDQEPAMLIDKV-VFTRLRPGKITDIIAQLKQGRSPAEIANPAGLPSQDIAYV   171

CmHoxF      ETPCIGLSDQEPAMLIDKV-VFTRLRPGKIADIIAQLKQGRSPAEIANPAGLPSDDIAYV   171

TxHoxF      TTSCTGLCDQGPALLVNGRA-MPRVTPERIDAMAGLIQQRVPVADW------PEDWFAIE   173

HpHoxF      RCSCTGLCDQGPALLVNHHQVVTRLDAGRVDQLAERLLAGVPPEDW------PADWFAVE   174

               * **.** **:*::    . *:   ::  :   :       :       * : :*

CnHoxF      DAMVESNVRTKGPVFFRGRTDLRSLLDQCLLLKPEQVIETIVDSRLRGRGGAGFSTGLKW   231

CmHoxF      DALVESNVRTKGPVFFRGRTDFKAVLDHCLTLRPEQVIDEIIESKLRGRGGAGFSTGLKW   231

TxHoxF      DRIH------RRDVLLRHVWRPGEVLQAARARGAQGVLNEVARSGLRGRGGAGFPTGFKW   227

HpHoxF      DHVR------RADVLLQGLPADAPSLPAVLSRTPAELLAEVERSGLRGRGGAGFPTARKW   228

            * :       :  *:::        *          ::  :  * ********* *.  **

CnHoxF      RLCRD--AESEQKYVICNADEGEPGTFKDRVLLTRAPKKVFVGMVIAAYAIGCRKGIVYL   289

CmHoxF      QLCRR--ALSDTKYVICNADEGEPGTFKDRALLTRSPKEVFIGMAIAAHAIGCRHGIVYL   289

TxHoxF      RACAE--APGAQRVIVCNADEGEPGTFKDRVLLSTAFDLVVDGMCVAALAVGARLGFLYL   285

HpHoxF      RACAEADAPDGQRCVVCNADEGEPGTFKDRVLLGRHADELFDGMTLAARAIGAQTGLVYL   288

            : *    * .  : ::**************.**    . :. ** :** *:*.: *::**

CnHoxF      RGEYFYLKDYLERQLQELREDGLLGRAIGGRAGFDFDIRIQMGAGAYICGDESALIESCE   349

CmHoxF      RAEYFYLKDYLERQLQQLRDDGLLGRAIGVRRDFDFDIRIQMGAGAYICGDESALIESCE   349

TxHoxF      RAEYRYLLPRLQARLAERRAQGLLGAGCLG-PGLDFDIDIHLGAGAYICGEESALIESLE   344

HpHoxF      RGEYRFLLPQLEAVLQRRRANGLLGRNAGGVAGFDFDIAIHLGAGAYVCGEESALIESLE   348
```

```
                *.** :*   *:  * . * :****       .:**** *::*****:**:******* *
CnHoxF          GKRGTPRVKPPFPVQQGYLGKPTSVNNVETFAAVSRIMEEGADWFRAMGTPDSAGTRLLS   409
CmHoxF          GKRGTPRVKPPFPVQQGYLGKPTCVNNVETFAAVSRIMEEGADWFRAMGTPDSAGTRLLS   409
TxHoxF          GKPGKPRIRPPFPVTQGYLGRPTTVNNVETLATAALVALHGGDWLRAIGTESSSGTKVLS   404
HpHoxF          GQRGTPRIRPPFPVQRGYLGRPTVVNNVETLVAVAHIARRGGAWWAGLGTPESTGTKIHS   408
                *: *.**::***** :****:** ******:.:.: :  .*. *  .:** .*:**:: *
CnHoxF          VAGDCSKPGIYEVEWGVTLNEVLAMVGARDARAVQISGPSGECVSVAKDGERKLAYEDLS   469
CmHoxF          VAGDCSKPGIYEVEWGVTLNEVLAMVGAKEARAVQISGPSGECVSVEKDGERRLAYEDLS   469
TxHoxF          VSGDVARPGIYECPFGTPLAEVLHAAGAADVQAVTTAGAAGPCLSAG-ELHRRIAFEDVA   463
HpHoxF          VSGDCERPGLYEYPLGTPLAQILADCGARNAQAVQVGGPSGSCVPAS-GFHRAIAFEDLP   467
                *:**  :**:**   *. * ::*   ** :.:**  .* :* *: .    .* :*:**:
CnHoxF          CNGAFTIFNCKRDLLEIVRDHMQFFVEESCGICVPCRAGNVDLHRKVEWVI----AGKAC   525
CmHoxF          CNGAFTIFNRNRDLLDIVKDYMQFFVDESCGICVPCRAGNVDLHRKVEWVI----AGKAC   525
TxHoxF          TGGSIMVFDRSRDLFELAHNVAHFFAHESCGFCTPCRVGTAVNARLLDKLAAH----RGS   519
HpHoxF          SAGALMVFDQTRDLFEVARHFARFFAHESCGLCTPCRVGTELVVRRLDKLAHERGAGRGS   527
                  *:: :*: .***:::.:.  :**..****:*.***.*.    * :: :       :..
CnHoxF          QKDLDDMVSWGALVRRTSRCGLGATSPKPILTTLEKFPEIYQNKLVRHEGPLLPSFDLDT   585
CmHoxF          QKDLDDMVSWGALVRKTSRCGLGATSPKPILTTLEKFPEIYQDKLVRHEGPLLPSFDLDT   585
TxHoxF          PYDLEEMDRMHRLMQGASHCGLGNTATLALQDLARKFRPAFERRLAS--TTYEPAFDLDA   577
HpHoxF          AFDIARLQELDALLHSGTHCGLGVSACNPLRDTLAHFGQAYAQRSTA--AQFQPGIDLDA   585
                  *:  :     *::  ::**** ::   :     :*   :  : .       *.:***:
CnHoxF          ALGGYEKALKDLEEVTR-------------    602
CmHoxF          ALGGHEKALKELEEAKK-------------    602
TxHoxF          ALSEARRMTRRDDALAHLGSNRSAGLGQTP    607
HpHoxF          ELSSARRATGRQDRGAHLNTEHGA------    609
                 *.  .:     :   :
```

HoxU Alignement

```
CnHoxU          MRYRMQEELIMSIQITIDGKTLTTEEGRTLVDVAAENGVYIPTLCYLKDKPCLGTCRVCS   60
CmHoxU          ----------MSIQITIDGITVTTEEGRTLVDVAAENGVYIPTLCYLKDKPCLGTCRVCS   50
TxHoxU          -----MSTPSASPTFLLDGQPVPFTPGQTVMQAAYAAGRYIAHLCWHPDFAAHGSCKLCT   55
HpHoxU          -----------MTTFDFDGQPVPLQPGDTILQAAQRAGHEVPHLCWHEGVSASASCRLCT   49
                              : :**  :    * *:::.*   *  :  **:  .  . .:*::*:

CnHoxU          VKVNGNVAAACTVRVSKGLNVEVNDPELVDMRKALVEFLFAEGNHNCPSCEKSGRCQLQA   120
CmHoxU          VKVNGNVNAACTVRVSKGLNVEVNSPEMVDMRKALVEFLFAEGNHNCPSCEKSGRCQLQA   110
TxHoxU          VQANGRLATGCTLAAAEGMEVRTQTPELEEKRRTLLQLLFVEGNHFCPACEKSGNCQLQA   115
HpHoxU          VVADGRPVPACATPAVAGQRVECHTEALKTRRLHLLQMLFVEGNHFCPGCEKSGNCQLQH   109
                * .:*.   .*:  .  * .*. :   :   *  *:::**.**** **.*****.****

CnHoxU          VGYEVDMMVSRFPYRFPVRVVDHASEKIWLERDRCIFCQRCVEFIRDKASGRKIFSISHR   180
CmHoxU          VGYEVDMMVSRFPYRFPVRVQDQASEKIWLERDRCIFCQRCVEFIRDKATGRKIFSISQR   170
TxHoxU          TAYEAGMLTPHFDHFFPDRPLDASHPDVLLDFNRCILCELCVRASR-DVDGKHVFALSGR   174
HpHoxU          QAERAGMTDLHYEPLHPERPVDASHPEVWFEPNRCILCQLCVRASD-ELDGKRVFAIGGH   168
                 . ...*   ::   .* *  * :  .: :: :***:*: **.    .  *:::*::. :

CnHoxU          GPESRIEIDAELANAMPP--EQVKEAVAICPVGTILEKRVGYDDPIGRRKYEIQSVRARA   238
CmHoxU          GSESRIEIDVELANAMPP--EQVKEAVAICPVGTILEKRVGYDDPIGQRKYEIQSVRARA   228
TxHoxU          GTQSHLIVNSPTGRLADTDFAATDRAADICPVGVILKKRVGFAVPIGQRRYDAQPASAVD   234
HpHoxU          GIGAKLLIDSESGRLGDSQLSVEDRAAHICPVGALLPKRVGFAQPYGQRTFDDAETRG--   226
                *  ::: ::   ..         ..*. *****.:* ****:  * *:* ::   . .

CnHoxU          LEGEDK---  244
CmHoxU          LGLEGVDK-  236
TxHoxU          VAPSSGGDA  243
HpHoxU          ---------  226
```

HoxY Alignement

```
CnHoxY      MTYAAEEELIMRAPHKDEIASHELPATPMDPAL---AANREGKIKVATIGLCGCWGCTLS   57

CmHoxY      ----------MSTAAKNELASHELPATPMDPAL---AANREGKIKVSMIGLCGCWGCTLS   47

TxHoxY      ----------------------------MNPPVDTPMATPPRKLKIATVSLAGCFGCHMS   32

HpHoxY      ----------------------------------MNTDSTPRKWRVATVSLAGCFGCHMS   26

                                                  .   * ::: :.*.**:** :*

CnHoxY      FLDMDERLLPLLEKVTLLRSSLTDIKRIPERCAIGFVEGGVSSEENIETLEHFRENCDIL   117

CmHoxY      FLDMDERLLPLLEKITILRSSLTDIKRIPERCAIGFVEGGVANEENIETLEHFRENCDIL   107

TxHoxY      FLDIDERLLPLLDLVEFDRSPLTDIKHC-GPCDIGLIEGGVCNAENVHVLREFRAQCRVL   91

HpHoxY      FLDIDERLFGLIEHITFDRSPLTDIKTV-GPCDIGLIEGGLCNAENVEVLRAFRDQCRVL   85

            ***:****: *:: : : ** *****     * **::***:.. **:..*. ** :* :*

CnHoxY      ISVGACAVWGGVPAMRNVFELKDCLAEAYVNSATAVPGAKAVVPFHPDIPRITTKVYPCH   177

CmHoxY      ISVGACAVWGGVPAMRNVVELKDCLAEAYVNSATAVAGAKAVIPFHPDIPRITTKVYPCH   167

TxHoxY      VALGACAVNGGLPAQRNHLAVGDVLQQVYITGHGLAPGS--QIPNDPELPLPLNQVHPVH   149

HpHoxY      VAVGACAITGGLPALRNHLDVGEMMKAVY---------G--EVPNDPELPLPLNRVRPIH   134

            :::****: **:** ** . : : :  .*         .   :* .*::*   .:* * *

CnHoxY      EVVKMDYFIPGCPPDGDAIFKVLDDLVNGRPFDLPSSINRYD      219

CmHoxY      EVVKMDYFIPGCPPDGDAIFKVLDDLVNGRPFDLPSSINRYD      209

TxHoxY      EVVRIDYFLPGCPPSGDAIWAFLNDLIAGREPRLGHGLLHYD      191

HpHoxY      EVVQIDHALPGCPPPADAFWQLLQDLMAGREPKLHKGLIRYD      176
            ***::*: :***** .**:: .*:**: **   *  .: :**
```

HoxH Alignement

```
CnHoxH      ---------------MSRKLVIDPVTRIEGHGKVVVHLDDDNKVVDAKLHVVEFRGFEKF  45

CmHoxH      ---------------MSRKLVIDPVTRIEGHGKVTVHLDDDNNVIDAKLHVVEFRGFEKF  45

TxHoxH      MTPAADLETAQVPREGLRRVAIDPVSRVEGHGKVTLLLDEQHRVHQVRLHIVEFRGFERF  60

HpHoxH      MTDAARPLETAADPQGLRRIVIDPVSRVEGHGKVTLLLDEQQRLQQVRLHIVEFRGFEQF  60

                             *::.****:*:******.: **:::.: :.:**:*******:*

CnHoxH      VQGHPFWEAPMFLQRICGICFVSHHLCGAKALDDMVGVGLKSGIHVTPTAEKMRRLGHYA  105

CmHoxH      IQGHPYWEAPMFLQRICGICFVSHHLCGAKALDDMVGVGLKSGIDVTPAAEKMRRLGHYA  105

TxHoxH      IQGRPYWEVPVMVQRLCGICPVSHHLAASKALDAVVG-----ARQLTPTAEALRRLMHYG  115

HpHoxH      IVGRPYWEVPVMVQRLCGICPVSHHLAASKALDRVVG-----GWPVPEAADRIRRLMQYG  115

            : *:*:**.*:::**:**** *****..:**** :**     .  :  :*: :*** :*.

CnHoxH      QMLQSHTTAYFYLIVPEMLFGMDAPPAQRNVLGLIEANPDLVKRVVMLRKWGQEVIKAVF  165

CmHoxH      QMLQSHTTAYFYLIVPEMLFGMDAPPEQRNVLGLIEADPELVKRVVMLRKWGQEVIKVVF  165

TxHoxH      QILQSHALHFFHLSSPDLLFGFESEVERRHLMGVAQAHPEIAKQGILLRKYGQEVIRLTS  175

HpHoxH      QIVQSHALHFFHLSSPDLLFGFDADVAQRNIVGVAMAHPEAARQGVMLRKFGQEVIRITS  175

            *::***:   :*:*  *::***:::   :*:::*:  *.*: .:: ::***:*****: .

CnHoxH      GKKMHGINSVPGGVNNNLSIAERDRFLNGEEGLLSVDQVIDYAQDGLRLFYDFHQKHRAQ  225

CmHoxH      GKKMHGINSVPGGVNKNLSIAERDRLLNGEEGLLAMDQVIDFAQDGLRLFYDFHEKHRAQ  225

TxHoxH      GKRVHGTASVPGGVNKALTGAERLALQQG------IGQVIDWSRQAVALVQRLHEQNPAL  229

HpHoxH      GKRVHGTGSVPGGMNRAVAREDRDTLRAQ------LPEVLAWAEAAVELAQRLHTGLPPA  229

            **::**  *****:*. ::  :*  :          : :*: ::. .: *   :*

CnHoxH      VDSFADVPALSMCLVGDDDNVDYYHGRLRIIDDDK-HIVREFDYHDYLDHFSEAVEEWSY  284

CmHoxH      VDSFADVPALNMSLVDADGNVDYYHGKLRIVDDDK-NIVRELDYHDYLDHFSEAVEEWSY  284

TxHoxH      YDTFGSFPSNFMGLVAPDGSLDLYDGALRATGADGQRLFDQFDVRGYDRLLTEAVKPWTY  289

HpHoxH      YEHFGETPAAMMSLIGPGGAMELYDGALRLREADGRIAVDGFEDQRYRELIDEAVKPWTY  289

             : *.. *:  * *:  .. :: *.* **    *    .  :: : *   : ***: *:*

CnHoxH      MKFPYLKELGREQGSVRVGPLGRMNVTKSLPTPLAQEALERFHAYTKGRT---NNMTLHT  341

CmHoxH      MKFPYLKDLGREKGSVRVGPLGRMNVTKTLSTPLAQEALERFHAYTKGRA---NNMTLHT  341

TxHoxH      MKFPYFTALGPEQGWYRVGPLARIQNADRLPTPLAEQARQQLLAYARARGHAMLHATLAY  349

HpHoxH      MKFPYRRALGPEAGWYRVGPLARLQNCDHIPTPRAEARRQAFV---AAHGGRPVHAVLAT  346

            *****   ** * *  *****.*::  . : ** *:   : :     .:     : .*
```

```
CnHoxH      NWARAIEILHAAEVVKELLHDPDLQKDQLVLTP-PPNAWTGEGVGVVEAPRGTLLHHYRA  400

CmHoxH      NWARAIEILHAAEVIRELLHDPDLQKDQLVLTP-PAGAWTGEGVGVVEAPRGTLLHHYRA  400

TxHoxH      HWARMIEMLHAAETIERLLHDDALEGTDLMTQGERPLAEVREGVGVIEAPRGTLIHHYQV  409

HpHoxH      HWARMIELLHGVEVIARLLDDPVILGGPLQATGER----QRSGVGIIEAPRGTLIHEYEV  402

            :*** **:**..*.: .**.*  :    *            .***::*******:*.*..

CnHoxH      DERGNITFANLVVATTQNNQVMNRTVRSVAEDYLGGHGEITEGMMNAIEVGIRAYDPCLS  460

CmHoxH      DERGNITFANLVVATTQNNQVMNRTVRSVAEDYLGGHGEITEGMMNAIEVGIRAYDPCLS  460

TxHoxH      GDDDLVTMANLIVSTTHNNQAMNTAVREVAQRYLDGR-ELTEGLLNHIEVAIRAYDPCLS  468

HpHoxH      GDDDLVKSCNLIVSTTHNNQAMNEAVRSVALQYLDGQ-TITEPLLNHLEVAIRAYDPCLS  461

            .: . :. .**:*:**:***.** :**.**  **.*:  :** ::* :**.*********

CnHoxH      CATHALGQMPLVVSVFDAAGRLIDERAR------------------- 488

CmHoxH      CATHALGQMPLVVSVYDAAGGLIDERTR------------------- 488

TxHoxH      CATHALGKMPLEVVLLDADGTELDRRLRPGGALLKP----------- 504

HpHoxH      CATHALGQMPLSVTLRGPDGEVLDHVLRSSTGETQRGATPHPMERAQ 508
            *******:*** * : .  *  :*.  *
```

HoxW Alignement

```
CnHoxW      MNAPAEFPYVTLADFDDPSTLIYGIGNVGRQDDGLGWAFI----DRLEAESLCSGAEVQR  56

CmHoxW      MNAPAEFPLVTLEDFDDPSSLIYGIGNVGRQDDGLGWALI----DWLEAESLCPKAEIQR  56

TxHoxW      ---------------MIAPTLILAWGNPSRGDDALGPLFAEAVQAW-----HLPGVECLT  40

HpHoxW      ------------MNEPVAPLLVLAWGNPSRGDDALGPMLAERLLAHAEAASLAGRVEVLT  48

                                *: . ** .* **.**  :                .*

CnHoxW      HYQLHLEDADLISRKRKVLFIDATKDAS--VASFSLERAEPRMDFSFTSHAISIPSIMAT  114

CmHoxW      HYQLHLEDADLISRKRRVLFIDATKDAS--VESFTLERAEPKMDFSFTSHAISIPSIMAT  114

TxHoxW      DFQLQVEHALDLQGRARVLFVDAARTPADHPGGYCVTALHPRRDASFTSHALSPPAVLQA  100

HpHoxW      DFQLQVEHALDLLGRERVLFVDAALDL---AEPFTVRPLQAARGGGIGSHALAPEAVLQV  105

            .:**::*.*  :  : :***:**:         : :   .   . .: ***::  ::: .

CnHoxW      CQRCFQCLP-EVYVLAIRGYEWELRMGLTPQARHNLDDAIAHFSMRAERQTS--------  165

CmHoxW      CQQCFQRLP-EVYVLAIRGYEWELKMGLTQRARQNLDDATAYLAMRRSVESREFS-----  168

TxHoxW      YADTLGTPPPA-WLLRLHGQRWGLGDDLSPPARAALAQALDWARDWLTPPARETIAPLQG  159

HpHoxW      YEDLHGHAPPASTLLAIRASAFELGAAPSVQALKDLEQAVAWVCRWLTGELVTEEVPA--  163

                    *    :* ::.  : *    :  *   * :*

CnHoxW      ---------   165

CmHoxW      ---------   168

TxHoxW      ATPEPASGR   168

HpHoxW      ---------   163
```
